# Chemotherapeutic Potential of Fluorouracil‐Platinum (IV) Prodrugs Against Cisplatin‐Resistant Colorectal Cancer Cells

**DOI:** 10.1002/chem.202503422

**Published:** 2026-02-27

**Authors:** Maria George Elias, Aleen Khoury, Shadma Fatima, Timothy J. Mann, Shawan Karan, Meena Mikhael, Paul de Souza, Christopher P. Gordon, Kieran F. Scott, Janice R. Aldrich‐Wright

**Affiliations:** ^1^ Faculty of Engineering Computing and Science Western Sydney University Sydney New South Wales Australia; ^2^ Faculty of Health School of Medicine Western Sydney University Sydney New South Wales Australia; ^3^ Medical Oncology Ingham Institute for Applied Medical Research Liverpool Hospital Liverpool New South Wales Australia; ^4^ Mass Spectrometry Facility Western Sydney University Sydney New South Wales Australia; ^5^ Nepean Clinical School Faculty of Medicine and Health University of Sydney Kingswood New South Wales Australia

**Keywords:** apoptosis, cytoskeleton, cytotoxicity, fluorouracil, platinum(II), platinum(IV)

## Abstract

Fluorouracil‐platinum(IV) prodrugs represent a novel class of multimechanistic chemotherapeutics with enhanced anticancer potential. The prodrugs **Pt^IV^P‐5FUMeOBut** and **Pt^IV^56‐5FUMeOBut** were actualized by derivatising the clinical drug 5‐fluorouracil (5FU) and coordinating it to platinum(IV) complexes, leveraging the established cytotoxicity of their platinum(II) precursors, **Pt^II^PHEN*SS*** and **Pt^II^56ME*SS***. The rationale behind this design is to combine the DNA synthesis‐disrupting antimetabolite activity of 5FU with platinum‐based cytotoxicity, aiming to enhance therapeutic efficacy. This study evaluates the in vitro potency of **Pt^IV^P‐5FUMeOBut** and **Pt^IV^56‐5FUMeOBut** in colorectal cancer models, assessing their cellular uptake, cytotoxicity, and effects in 3D spheroid cultures. Comparative analyses with cisplatin and 5FU explore their impact on cell survival, reactive oxygen species production, mitochondrial membrane potential, and cell‐cycle progression. Mechanistic insights are further examined through apoptosis and necrosis assays, proteomic profiling, and western blot analysis of key signalling pathways. These findings contribute to the preclinical development of fluorouracil‐platinum(IV) prodrugs as potential candidates for cancer therapy.

## 1 Introduction

Colorectal cancers (CRC), encompassing both colon and rectal cancers, rank second in cancer‐related mortality and are the third most diagnosed cancers in the United States [1, 2]. Early‐stage colon cancer is typically treated with surgery, often followed by adjuvant chemotherapy using agents such as fluorouracil and cisplatin. However, recurrence and resistance to these drugs remain major clinical challenges [2, 3, 4, 5, 6, 7, 8, 9]. Overcoming resistance to standard treatments like cisplatin is essential to improving therapeutic efficacy and extending patient survival.

Medicinal inorganic chemistry leverages the unique properties of metals for disease diagnosis and treatment. Metal ions, which readily form cations, can interact with negatively charged biological molecules, enabling charge modulation and hydrolysis reactions [10, 11, 12, 13]. These characteristics have spurred growing interest in developing metal‐based anticancer agents through this discipline [12, 14]. The beginnings of modern metal‐based chemotherapy date back to the year 1844, when Michele Peyrone synthesized *cis*‐diamminedichloroplatinum(II) (cisplatin), although its anticancer potential was not recognized until 1965 [15]. Despite the clinical success, the widespread use of cisplatin has been restricted by challenges related to resistance and toxicity. To overcome these limitations, second‐ and third‐generation platinum drugs were introduced, notably *cis*‐diammine(1,1‐cyclobutanedicarboxylato)platinum(II) (carboplatin) and *trans*‐*L*‐(1*R*,2*R*‐diaminocyclohexane)oxalatoplatinum(II) (oxaliplatin) [16]. Carboplatin received FDA approval in 1989 for the treatment of head and neck, lung, and ovarian cancers [17], while oxaliplatin was approved in 2002 for advanced colorectal cancer [18]. Ongoing research continues to focus on developing next‐generation platinum‐based drugs with improved efficacy and reduced toxicity and resistance [16, 19, 20, 21, 22].

The development of novel platinum coordination complexes suitable for oral delivery has accelerated, driven in part by concerns over the poor solubility and limited bioavailability of clinically used platinum(II) complexes [23, 24, 25, 26]. Platinum(IV) complexes have been developed as promising options, due to their octahedral six‐coordinate geometry, which features two axial positions and enhanced kinetic inertness. Due to these characteristics, platinum(IV) complexes are considered promising prodrug candidates, as they can reduce off‐target interactions [20, 27]. These complexes are engineered to remain chemically stable in the bloodstream while undergoing bio‐reductive activation in the presence of intracellular reductants such as ascorbic acid and glutathione, thereby enhancing their chances of reaching intended biological targets [20, 24, 28, 29, 30, 31, 32]. Continued progress in explicating resistance mechanisms, tumor uptake, and structure–activity relationships will be critical to advancing the next generation of platinum‐based therapies [16, 33, 34]. Satraplatin, chemically known as *bis*‐(acetate)‐amine‐dichloro‐(cyclohexylamine)platinum(IV), progressed to phase III clinical trials, targeting hormone‐refractory prostate cancer but failed to show a meaningful increase in survival rates [28, 35, 36]. Platinum(IV) prodrugs with bioactive axial ligands have been developed to selectively bind specific enzymes or polypeptides, as highlighted in recent literature [37, 38]. However, little progress has been made in conjugating platinum with axial ligands that function as antimetabolites (pyrimidine analogs), such as 5‐Fluorouracil (Fluorouracil, marketed as Adrucil) [39, 40, 41]. Ongoing research into platinum prodrugs not only broadens the therapeutic landscape but also informs the design of future treatment strategies, enabling the development of novel drug combinations and therapeutic regimens [16, 42, 43].

**FIGURE 1:**
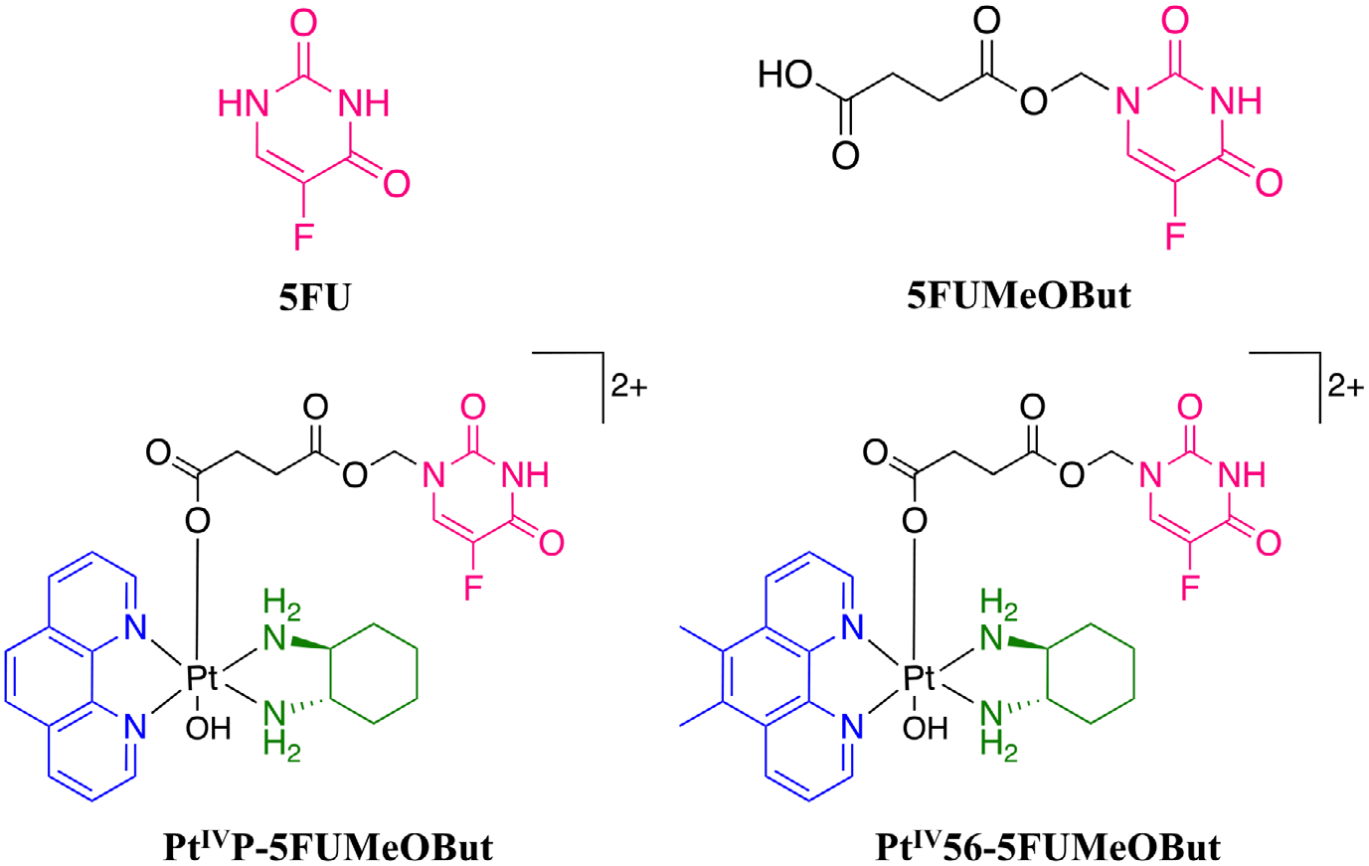
Structures of 5FU, 5FUMeOBut and fluorouracil‐platinum(IV) prodrugs (ChemDraw 22.0.0), Nitrate (NO_3_)_2_ counterions were omitted for clarity.

A series of platinum(II) and platinum(IV)‐dihydroxy precursor complexes have been synthesised and characterised by our team that exhibit promising anticancer activity across various cancer models [39, 44]. In this study, we investigate the anticancer potential of novel platinum(IV) prodrugs derived from our non‐DNA‐coordinating platinum(II) scaffolds [Pt(1,10‐phenanthroline)(1*S*,2*S*‐diaminocyclohexane)](NO_3_)_2_ (**Pt^II^PHEN*SS***) and [Pt(5,6‐dimethyl‐1,10‐phenanthroline)(1*S*,2*S*‐diaminocyclohexane)](NO_3_)_2_ (**Pt^II^56ME*SS***) in conjugation with the antimetabolite 5‐fluorouracil (5FU) (see Figure 1). The core platinum(II) complexes follow the general formula [Pt^II^(H_L_)(A_L_)]^2+^, where H_L_ is the heterocyclic ligand (1,10‐phenanthroline or 5,6‐dimethyl‐1,10‐phenanthroline) and A_L_ is the ancillary ligand (1*S*,2*S*‐diaminocyclohexane (*S*,*S*‐DACH)**)**.

Accordingly, it was theorized that forming fluorouracil‐platinum(IV) prodrugs would enhance the anticancer effect, compared to 5FU, cisplatin or the core scaffold platinum(II) complexes, consistent with previous findings that functionalizing platinum(IV) complexes with pharmacologically active axial ligands can improve therapeutic efficacy [24, 39, 41]. The resulting, fluorouracil‐platinum(IV) prodrugs would exhibit a multimechanistic action as previously observed as well as exhibiting the added mechanism of targeting DNA synthesis with 5FU [39, 44]. 5FU inhibits the thymidylate synthase (TS) enzyme, which is crucial for the formation of thymidylate, an essential precursor for DNA biosynthesis, making it a critical target in cancer therapy [39, 45, 46, 47]. Therefore, such a prodrug combines cytoskeletal, protein and DNA targeting properties in a single entity. It is worth noting the coadministration of 5FU with other chemotherapeutics has been reported to increase its sensitivity to resistant cancers particularly colorectal cancer [40, 48]. Accordingly, methoxybutanoic acid was employed as a cleavable linker to enable intracellular release of 5FU. Integration into a platinum(IV) scaffold is expected to enhance anticancer activity, particularly in resistant colorectal cancer [39].

In this context, we examine the biological activity of two novel 5FU‐conjugated platinum(IV) prodrugs: [Pt(1,10‐phenanthroline)(1*S*,2*S*‐diaminocyclohexane)(5‐fluorouracil‐1‐methoxy‐4‐oxobutanate)(dihydroxide)] nitrate (**Pt^IV^P‐5FUMeOBut**), and [Pt(5,6‐dimethyl‐1,10‐phenanthroline)(1*S*,2*S*‐diaminocyclohexane)(5‐fluorouracil‐1‐methoxy‐4‐oxobutanate)(dihydroxide)] nitrate (**Pt^IV^56‐5FUMeOBut**), as illustrated in Figure 1. Upon bioreductive activation, these prodrugs are designed to release the active platinum(II) forms and exert anticancer effects through disruption of the microtubule network, induction of reactive oxygen species (ROS), mitochondrial dysfunction, and metabolic dysregulation, ultimately triggering cell death [44, 49, 50].

Building on our previous study, which established the synthesis and broad anticancer activity of 5FU‐conjugated platinum(IV) complexes across multiple cancer cell lines, the present work specifically focuses on elucidating their mechanism of action in cisplatin‐resistant colorectal cancer cells (HT29) [39]. HT29 cells were explicitly selected owing to their well‐documented resistance to cisplatin and the pronounced sensitivity observed for these 5FU‐conjugated platinum(IV) prodrugs in earlier screening. This in our estimation made them a relevant model for mechanistic investigation. Accordingly, this study is designed to provide an in‐depth biological characterization encompassing cellular uptake and localization, oxidative stress, mitochondrial dysfunction, cell‐cycle disruption, and cell‐death pathways using complementary biochemical, imaging, and proteomic approaches.

This investigation addresses a critical need in oncology by targeting chemoresistance in colorectal cancer and builds on current knowledge of platinum drug pharmacology. A better understanding of these prodrugs could contribute significantly to the development of more effective treatments. Progression to in vivo studies will be determined by their in vitro performance.

## 2 Results

### 2.1 Cytotoxicity Evaluation of Fluorouracil‐Platinum(IV) Prodrugs in Colorectal Cancer Cells, Normal Epthelial Cells, and Spheroid Model of Colorectal Cancer

After 72 h of treatment, the cytotoxic effects of the 5FU‐platinum(IV) prodrugs were assessed in HT29 colon cancer cells, 3D spheroids, and in MCF10A cells (immortalized, nontumorigenic human breast epithelial cell line) (Figure S1 and Table 1). For context, the IC_50_ values of the corresponding platinum(II) complexes (**Pt^II^PHEN*SS*** and **Pt^II^56ME*SS***) [44], which represent the expected intracellular reduction products of the platinum(IV) prodrugs, are also reported in Table 1. All prodrugs showed exceptional potency when compared to cisplatin and the ligands 5FU or 5FUMeOBut. The potency relative to cisplatin was highest for **Pt^IV^56‐5FUMeOBut** (707‐fold, *p* < 0.0001) compared to **Pt^IV^P‐5FUMeOBut** (93‐fold, *p* < 0.0001). The ligands, 5FU or 5FUMeOBut were respectively 10‐ and 5‐fold more potent than cisplatin (*p* < 0.0001). Importantly, the IC_50_ values of the platinum(II) species are approximately 10–90‐fold more potent than the corresponding to 5FU and its derivatives, indicating that platinum(II) dominates the overall cytotoxicity at cytotoxic concentrations. As expected, the precursor platinum(II) complexes displayed higher intrinsic cytotoxicity than the corresponding platinum(IV) conjugates, consistent with the requirement for intracellular reduction prior to activation.

**TABLE 1:** IC_50_ values (µM) of 5FU, 5FUMeOBut, **Pt^IV^P‐5FUMeOBut** or **Pt^IV^56‐5FUMeOBut** prodrugs, and cisplatin on HT29, HT29 spheroids, and MCF10A cell lines.

IC_50_ (μ M)	Cisplatin	Pt^II^PHEN*SS*	Pt^II^56ME*SS*	5FU	5FUMeOBut	Pt^IV^P‐5FUMeOBut	Pt^IV^56‐5FUMeOBut
**MCF10A**	4.71 ± 1.47^a^	3.49 ± 1.39	0.41± 1.52	141.9 ± 2.01 ****^b^	171.00 ± 3.86 ****	4.33 ± 1.35	3.63 ± 1.59
**HT29**	77.76 ± 1.97	0.76 ± 1.47 ****	0.18 ± 1.49 ****	7.57 ± 1.24 ****	15.68 ± 1.29 ****	0.84 ± 1.11 ****	0.11 ± 1.13 ****
**HT29 Spheroids**	444.8 ± 5.09	3.93 ± 1.46 ****	0.99 ± 1.29 ****	43.30 ± 2.05 ****	49.70 ± 1.62 ****	8.67 ± 1.44 ****	2.41 ± 1.19 ****

The data for cisplatin, **Pt^II^PHEN*SS*** and **Pt^II^56ME*SS*** was obtained from previously published data run in parallel [44].

^a^ Data are mean ± SEM of three separate experiments.

^b^ Significance of IC_50_s compared to cisplatin are identified by **** *p* < 0.0001, as measured by an unpaired Student's t‐test. NS = No Significance.

To investigate the cytotoxic effects of the prodrugs in a system that more closely mimics in vivo drug responses, spheroids from the HT29 cell line were bio‐printed, cultured, and treated for 72 h (Figures S1 and S2) [51]. The overall cytotoxicity decreased in 3D spheroids; however, the order of potency among the prodrugs remained consistent. **Pt^IV^P‐5FUMeOBut** and **Pt^IV^56‐5FUMeOBut** were more potent than cisplatin, 5FU or 5FUMeOBut. The higher potency of the platinum(II) complexes relative to the platinum(IV) prodrugs was consistent in 3D spheroids, further supporting their role as the primary cytotoxic species released upon intracellular reduction. In HT29 spheroids, the potency of the prodrugs relative to cisplatin was greatest for **Pt^IV^56‐5FUMeOBut** (185‐fold, *p* < 0.0001) followed by **Pt^IV^P‐5FUMeOBut** (51‐fold, *p* < 0.0001) > 5FU (10‐fold, *p* < 0.0001) > 5FUMeObut (9‐fold, *p* < 0.0001). The IC_50_ values increased in 3D HT29 spheroid culture compared to 2D culture with an average 9‐fold increase. IC_50_ values for **Pt^IV^P‐5FUMeOBut** differed significantly (*p* < 0.05) between 2D and 3D growth conditions. The substantial differences in intrinsic IC_50_ values between the platinum(II) species and 5FU indicate that a 1:1 combination of the components would not be expected to significantly alter the observed IC_50_ trends of the prodrugs. However, IC_50_ values for **Pt^IV^P‐5FUMeOBut** differed significantly between 2D and 3D growth conditions.

The MCF10A cell line, derived from nontumorigenic human mammary tissue, is widely used as a “normal” control in cytotoxicity studies due to its epithelial morphology and expression of both epithelial and luminal markers [52, 53, 54]. It serves as a baseline for comparing drug effects on noncancerous versus cancerous cells and is commonly used to calculate the selective cytotoxicity index (SCI) or therapeutic index [52, 55, 56, 57, 58, 59, 60, 61, 62, 63, 64]. The SCI (Table 2) was determined by dividing each drug's half‐maximal inhibitory concentration (IC_50_) in MCF10A cells by its IC_50_ in a cancer cell line (Table 1). The evaluated prodrugs demonstrated selectivity in HT29 (cisplatin‐resistant) cells, with an SCI greater than 5, compared to the clinical platinum drug cisplatin, which showed an SCI of 0.06. Considering this, the selectivity was highest for **Pt^IV^56‐5FUMeOBut** (SCI of 33), followed by 5FU (SCI of 18.75) > 5FUMeOBut (SCI of 10.91) > **Pt^IV^P‐5FUMeOBut** (SCI of 5.15). Since HT29 is a cisplatin‐resistant cell line, it was anticipated that cisplatin would exhibit the lowest SCI.

**TABLE 2:** Comparison of the selective cytotoxicity index of 5FU, 5FUMeOBut, their Pt^IV^ prodrugs (**Pt^IV^P‐5FUMeOBut** and **Pt^IV^56‐5FUMeOBut**), and cisplatin in cancer cells.

^a^SCI	Cisplatin	Pt^II^PHEN*SS*	Pt^II^56ME*SS*	5FU	5FUMeOBut	Pt^IV^P‐5FUMeOBut	Pt^IV^56‐5FUMeOBut
**HT29**	0.06	4.59	2.28	18.75	10.91	5.15	33

^a^ SCI = IC_50_ (normal epithelial cell line, MCF10A)/IC_50_ (cancer cell line).

### 2.2 Cellular Uptake of the Fluorouracil‐Platinum(IV) Prodrugs

**FIGURE 2:**
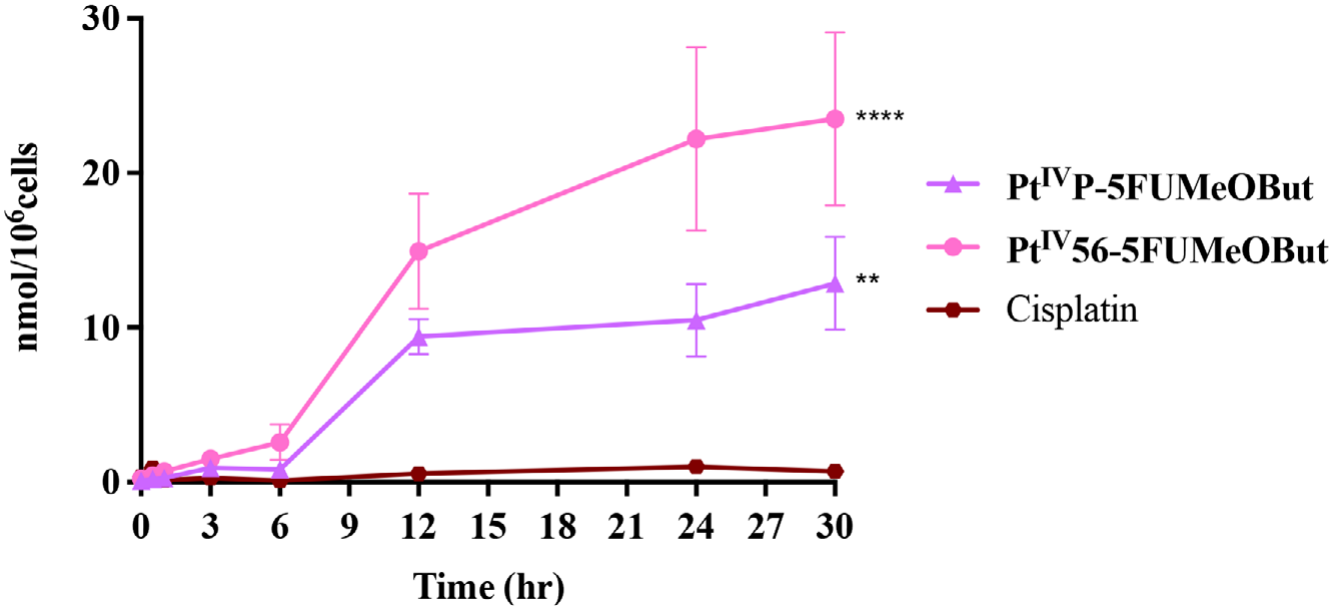
Cellular uptake of **Pt^IV^P‐5FUMeOBut** and **Pt^IV^56‐5FUMeOBut**. ICP‐MS was used to quantify platinum uptake in HT29 cells at 0, 0.5, 1, 3, 6, 12, 24, and 30 h, following the procedure outlined in Method 5.5. Cisplatin values were derived from previously published experiments performed in parallel [44]. Results are presented as mean ± SEM (*n* = 3) from three independent experiments, with each sample analysed in triplicate and expressed as nmol/10^6^ cells. Statistical significance relative to cisplatin was determined by two‐way ANOVA (**p < 0.01, ****p < 0.0001).

The cellular absorption of Pt in cells treated with **Pt^IV^P‐5FUMeOBut** and **Pt^IV^56‐5FUMeOBut** relative to cisplatin, was determined by ICP‐MS at a concentration of 3 µM for each treatment. In HT29 cells, cisplatin showed the lowest level of cellular uptake compared with the prodrugs. Uptake of both prodrugs (Figures 2, S3 and S4) increased in a time‐dependent manner, with a marked rise between 6–12 h and a steady accumulation observed up to 30 h. Cisplatin cell uptake remained relatively stable, with a decrease in intracellular concentrations observed from 24 to 30 h. The highest mean intracellular concentrations (nmol/10^6^ cells) were observed in HT29 cells for **Pt^IV^56‐5FUMeOBut** (0.25 ± 0.08 (0 h) to 23.51 ± 5.61 (30 h)) > **Pt^IV^P‐5FUMeOBut** (0.12 ± 0.05 (0 h) to 12.87 ± 3.01 (30 h)) > cisplatin (0.36 ± 0.04 (0 h) to 0.71 ± 0.18 (30 h)) (Table S3).

The enhanced uptake of **Pt^IV^56‐5FUMeOBut** could be attributed to the increased hydrophobic interactions with biomolecules, facilitated by the presence of the methyl groups, which promote cellular uptake [65]. Cellular volume is approximately 1.7 pL [66]. With the complexes evenly distributed inside the cell, the drug concentration was determined to be around 2022 µM and 3693 µM per HT29 cell for **Pt^IV^P‐5FUMeOBut** and **Pt^IV^56‐5FUMeOBut**. This corresponds to a 674‐fold and 1231‐fold increase compared to the starting concentration of 3 µM for **Pt^IV^P‐5FUMeOBut** and **Pt^IV^56‐5FUMeOBut**, respectively. The ratio (intracellular/extracellular concentration) was estimated from the extracellular concentration of 3 µM for the platinum complexes (Figure 2 and Table S3). Both prodrugs showed a significant accumulation across all time points. At 30 h, **Pt^IV^56‐5FUMeOBut** recorded the greatest intracellular/extracellular accumulation ratio in HT29 cells (3693 ± 2640); a result which corresponds to a 33‐fold higher maximum ratio than that reported for cisplatin (with a ratio of 112 ± 83 at 30 h of treatment).

The increase in cellular concentrations suggest an active mode of transport for cell uptake, similar to cisplatin, which enters cells via both active transport through membrane proteins like CTR1 and passive diffusion [67]. The comparative uptake of the complexes, relative to cisplatin and to one another, was evaluated using a two‐way ANOVA, with time as the first factor and the uptake response of each complex as the second factor. In HT29 cells, the uptake of **Pt^IV^P‐5FUMeOBut** and **Pt^IV^56‐5FUMeOBut** were all significant relative to cisplatin with *p* < 0.01 and *p* < 0.0001, respectively.

### 2.3 Mode of Uptake of Fluorouracil‐Platinum(IV) Prodrugs

**FIGURE 3:**
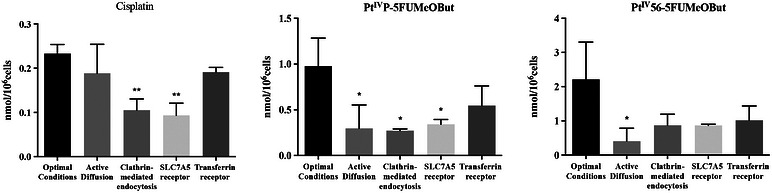
Mode of cellular uptake of platinum in HT29. ICP‐MS was employed to quantify intracellular platinum following incubation at either 37°C or 4°C, and after inhibition of SLC7A5, the transferrin receptor, or clathrin‐mediated endocytosis, as outlined in Method 5.6. Cisplatin data was obtained from previously reported data run in parallel [44]. Data denote mean ± SEM of *n* = 3 from three separate experiments where samples were run in triplicates and expressed in nmol/10^6^ cells. *p < 0.05 and ** *p* < 0.01 compared to optimal conditions, as measured by one‐way ANOVA.

To determine if any essential transport proteins or uptake mechanisms are involved in the intracellular absorption of the prodrugs, the cellular uptake of **Pt^IV^P‐5FUMeOBut** and **Pt^IV^56‐5FUMeOBut** was measured using ICP‐MS after inhibiting the mechanism of interest (Figure 3 in nmol/10^6^ cells and Figure S4 in 𝜇M/cell). The cellular uptake of cisplatin was slightly decreased at 4°C (which affects active diffusion) compared to the optimal condition of 37°C. However, inhibiting clathrin‐mediated endocytosis or SLC7A5 led to a significant reduction in uptake (*p* < 0.01) in HT29 cells. Additionally, the uptake of TfR was also slightly reduced upon inhibition. HT29 cells treated with **Pt^IV^P‐5FUMeOBut** exhibited a significant reduction in uptake at 4°C, for the clathrin‐mediated endocytosis and SLC7A5 receptor upon inhibition (*p* < 0.05) and a decrease in uptake on TfR inhibition. A significant reduction in **Pt^IV^56‐5FUMeOBut** uptake was observed in cells maintained at 4°C (*p* < 0.05) whereas inhibition of clathrin‐mediated endocytosis, SLC7A5 receptor, and TfR, caused a decreasing trend that was not statistically significant.

### 2.4 Cellular Localization of Fluorouracil‐Platinum(IV) Prodrugs

**FIGURE 4:**
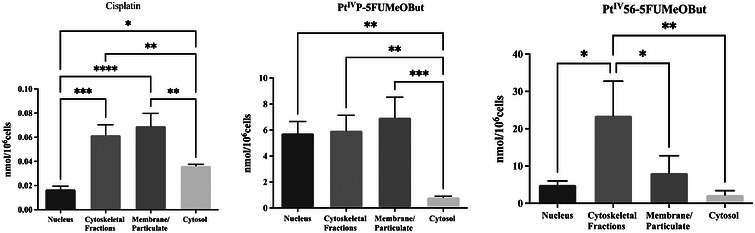
Cellular localization of platinum in HT29 post 24 h. The amount of intracellular platinum was determined by ICP‐MS after cellular fractionation, according to Method 5.7. Data for cisplatin were obtained from previously reported experiments run in parallel [44]. Each value represents the mean ± SEM of three independent determinations, with samples run in triplicate and expressed as nmol/10^6^ cells. One‐way ANOVA was used to assess statistical differences between fractions (*p < 0.05, **p < 0.01, ***p < 0.001 and ****p < 0.0001).

The intracellular accumulation of the prodrugs was investigated for localization in the nuclear, cytoskeletal, membrane/particulate, and cytosolic fractions of HT29 cells by ICP‐MS (Figures 4 and S6). The bar graphs illustrate the amount of platinum in each fraction. These data represent relative enrichment of platinum in the different fractions rather than absolute organelle‐specific concentrations and are intended to provide comparative insight into the intracellular localization of the prodrugs. **Pt^IV^56‐5FUMeOBut** was primarily concentrated in the cytoskeletal fraction, whereas **Pt^IV^P‐5FUMeOBut** was mainly localized to the membrane/particulate, cytoskeletal, and nuclear fractions. In HT29 cells, cisplatin accumulated predominantly in the membrane/particulate fraction (*p* < 0.0001 vs nucleus) and the cytoskeletal fraction (*p* < 0.001 vs nucleus), consistent with previous observations in ovarian cancer cells [68]. Although related platinum complexes have been reported to predominantly accumulate in the cytoskeleton [44, 50, 69], **Pt^IV^56‐5FUMeOBut** was significantly localized in cytoskeleton compared to the nucleus (*p* < 0.05), membrane/particulate (*p* < 0.05) and cytosol fraction (*p* < 0.01). This finding aligns with observations from the platinum(II) precursors and platinum(IV) derivatives of the prodrugs, suggesting that a similar mechanism of action may be involved [44]. **Pt^IV^P‐5FUMeOBut** was the most localized to the membrane/particulate in comparison to the cytosol (*p* < 0.001) > the cytoskeletal fraction (*p* < 0.01) > nuclear fraction (*p* < 0.01).

### 2.5 Cell Death Analysis

**FIGURE 5:**
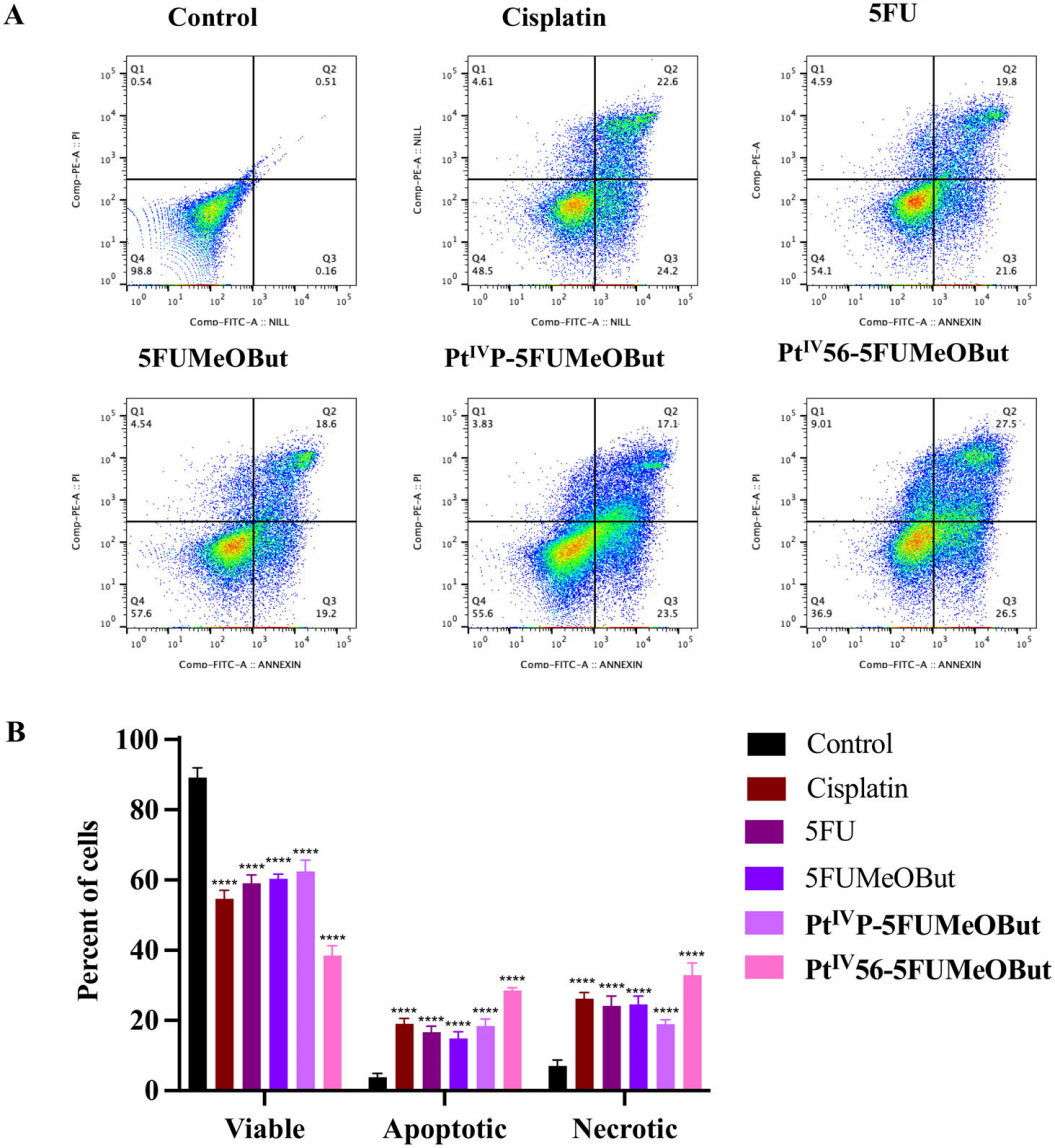
Flow cytometric analysis of cell death mediated by cisplatin, 5FU, 5FUMeOBut, and the prodrugs, **Pt^IV^P‐5FUMeOBut** or **Pt^IV^56‐5FUMeOBut**. HT29 cells were treated with the complex and analyzed at 72 h as described in Method 5.8. (A). Representative dot plots and (B). Bar graphs representing percent viable, apoptotic, and necrotic cells. Data points denote mean ± SEM of *n* = 3 from three separate determinants where samples were run in triplicates. **** *p* < 0.0001 compared to control group, as measured by one‐way ANOVA.

Annexin V/PI staining was used to determine the type of cell death. HT29 cells were treated with **Pt^IV^P‐5FUMeOBut** and **Pt^IV^56‐5FUMeOBut** prodrugs, 5FU, 5FUMeOBut, with cisplatin was used as a positive control (Figure 5). Flow cytometry was measured at 72 h post treatment. All investigated agents, including cisplatin, exhibited significant (p < 0.0001) early apoptosis and late apoptosis/necrosis (Figure 5B).

### 2.6 Cell Cycle Arrest

**FIGURE 6:**
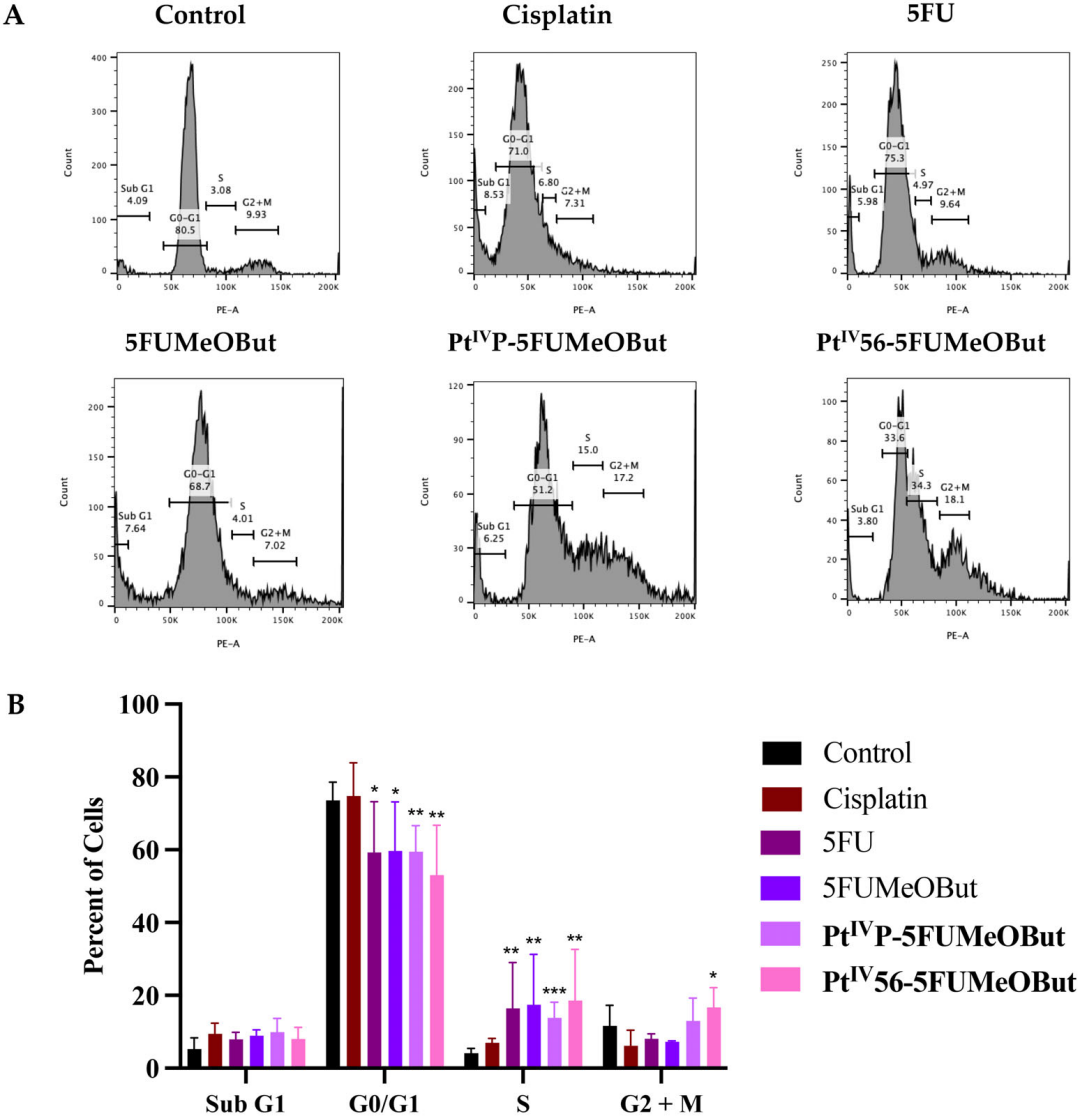
Flow cytometric analysis of cell cycle mediated by cisplatin, 5FU, 5FUMeOBut, **Pt^IV^P‐5FUMeOBut** and **Pt^IV^56‐5FUMeOBut**. HT29 cells were treated with IC_30_ concentration of each complex and analysed at 72 h as described in Method 5.9. A representative histogram plots and B. Bar graphs representing percent Sub G1, G0/G1, S, and G2+M phases of cell cycle. Data points denote mean ± SEM of *n* = 3 from three separate experiments where samples were run in triplicates. * *p* < 0.05, ** *p* < 0.01, and *** *p* < 0.001 relative to control group, as measured by one‐way ANOVA.

The cell‐cycle profiles of HT29 cells treated for 72 h with the prodrugs, **Pt^IV^P‐5FUMeOBut** and **Pt^IV^56‐5FUMeOBut**, 5FU, 5FUMeOBut or cisplatin, at concentrations equivalent to their IC_30_ values was studied (Figure 6). In HT29 treated cells, an increase in the proportion of cells in the S phase of the cell cycle was observed with all ligands (*p* < 0.01) and prodrugs (**Pt^IV^P‐5FUMeOBut**; *p* < 0.001 and **Pt^IV^56‐5FUMeOBut**; *p* < 0.01) (Figure 6B). This was similarly observed by their platinum(II) precursors [44]. A significant increase in G2+M phase of the cell cycle was detected in **Pt^IV^56‐5FUMeOBut** (*p* < 0.05) treatment. In HT29 cells, cisplatin was mainly concentrated in the G0/G1 and S phases of the cell cycle, showing higher levels compared to the control cells.

### 2.7 Reactive Oxygen Species (ROS)

**FIGURE 7:**
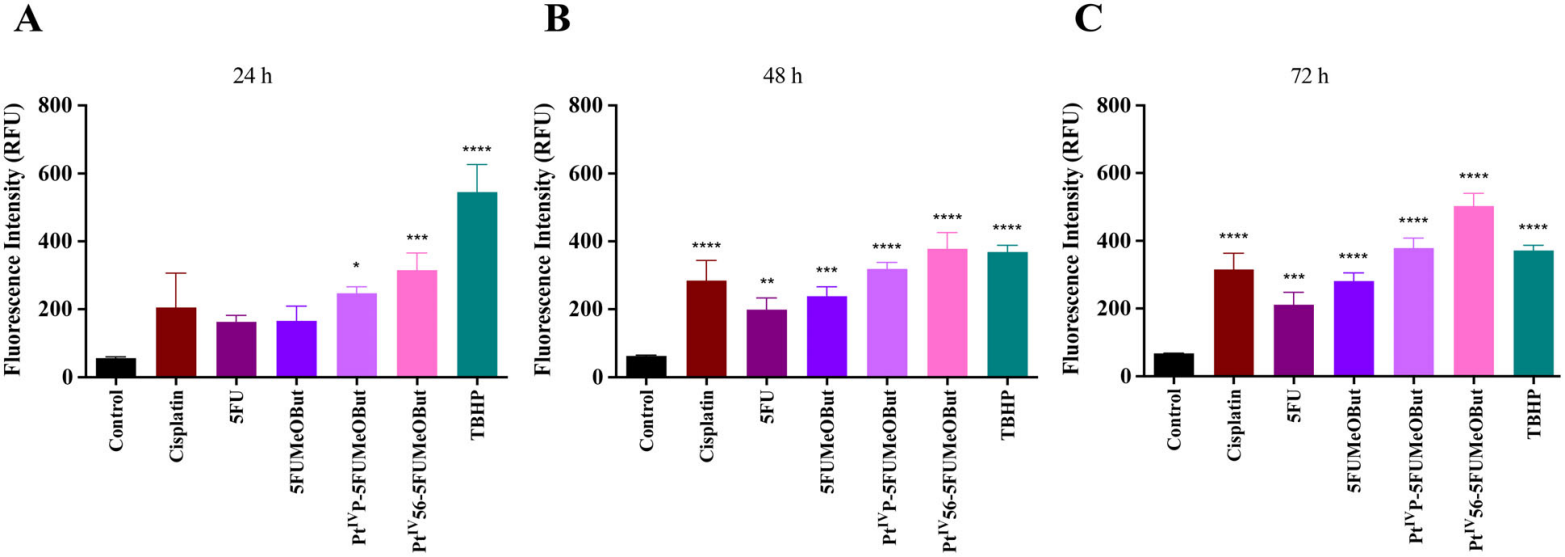
ROS production upon treatment with **Pt^IV^P‐5FUMeOBut** or **Pt^IV^56‐5FUMeOBut** prodrugs, 5FU, 5FUMeOBut, cisplatin and TBHP were measured in HT29 cells at 24, 48 and 72 h as described in Method 5.10. * *p* < 0.05, ** *p* < 0.01, *** *p* < 0.001, and **** *p* < 0.0001 relative to control. Cisplatin data was obtained from previously reported data run in parallel [44]. Data points denote mean ± SEM of *n* = 3 from three separate experiments where samples were run in triplicate.

HT29 cells were treated with **Pt^IV^P‐5FUMeOBut** and **Pt^IV^56‐5FUMeOBut** prodrugs, 5FU, 5FUMeOBut or cisplatin using individual IC_50_ value concentrations to study the production of ROS. Upon the production of ROS, DCFDA produces a fluorescent product, which is proportional to the produced ROS (Figures 7, S7, and Table S4). HT29 cells treated with prodrugs **Pt^IV^P‐5FUMeOBut** and **Pt^IV^56‐5FUMeOBut** exhibited a notable increase in ROS starting at 24 h (*p* < 0.05 and *p* < 0.001), through to 48 and 72 h (*p* < 0.0001). 5FU, 5FUMeOBut and cisplatin exhibited increased ROS at 48 and 72 h (*p* < 0.0001). TBHP, the positive control produced the most ROS at 24 h (*p* < 0.0001) and while this decreased, it was still significant at 48 and 72 h with *p* < 0.0001. Notably, the 5FU‐platinum(IV) prodrugs induced higher ROS levels than their corresponding platinum(II) precursors at all time points (Table S4), despite the coreleased 5FU being present at sub‐cytotoxic concentrations. A significant difference in ROS production was observed by **Pt^IV^P‐5FUMeOBut** relative to **Pt^IV^56‐5FUMeOBut** (*p* < 0.01) at 72 h. A significant increase in ROS produced by **Pt^IV^56‐5FUMeOBut** relative to cisplatin (*p* < 0.0001) was observed at 72 h, as measured by one‐way ANOVA.

### 2.8 Mitochondrial Membrane Potential

**FIGURE 8:**
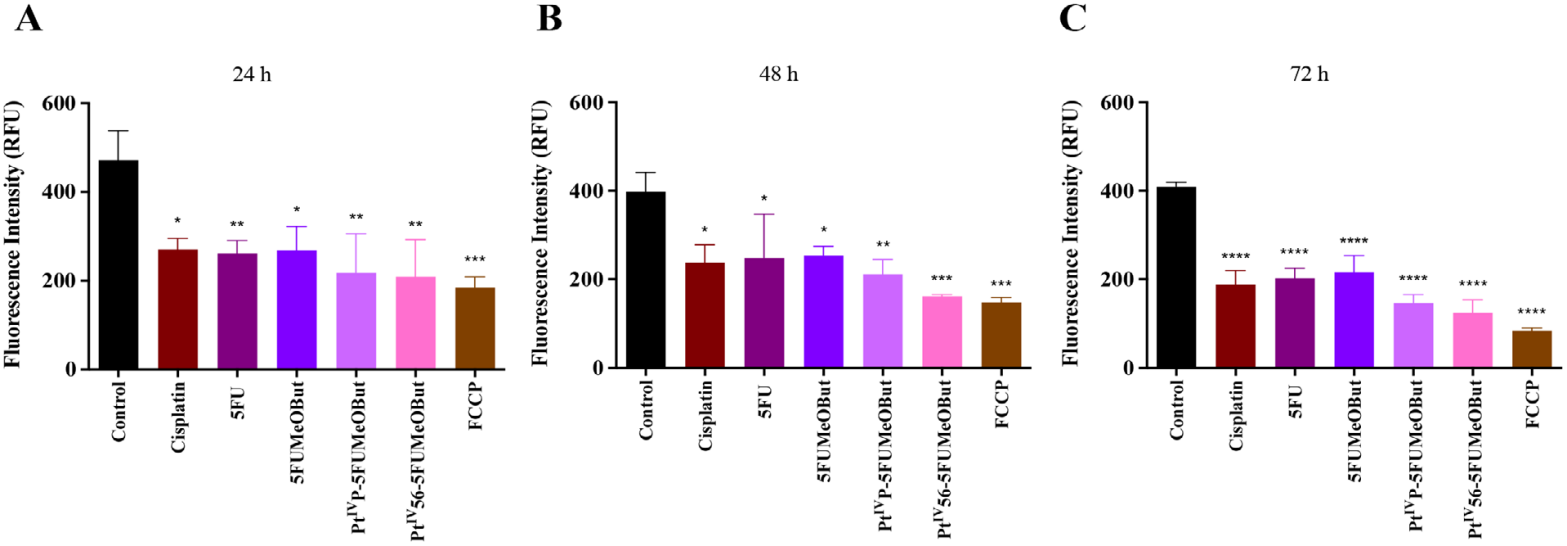
Changes in MtMP following treatment with **Pt^IV^P‐5FUMeOBut**, **Pt^IV^56‐5FUMeOBut**, 5FU, 5FUMeOBut, cisplatin, or FCCP were assessed in HT29 cells at 24, 48, and 72 h, as described in Method 5.11. Cisplatin data were obtained from previously reported parallel experiments [44]. Data points denote mean ± SEM of *n* = 3 from three separate experiments where samples were run in triplicate. * *p* < 0.05, ** *p* < 0.01, *** *p* < 0.001, and **** *p* < 0.0001 compared with control, as measured by one‐way ANOVA.

HT29 cells were treated with **Pt^IV^P‐5FUMeOBut** and **Pt^IV^56‐5FUMeOBut** prodrugs, 5FU, 5FUMeOBut or cisplatin using individual IC_50_ value concentrations to study mitochondrial membrane potential (MtMP). TMRE was used to measure relative fluorescence units (RFU), which are proportional to MtMP changes (Figure 8 and Table S5). Treatment with **Pt^IV^P‐5FUMeOBut** and **Pt^IV^56‐5FUMeOBut** resulted in a pronounced decrease in MtMP beginning at 24 h with significant reductions maintained at 48 h and further decreased at 72 h. Cisplatin, 5FU, and 5FUMeOBut also induced significant hypopolarization at 72 h (*p* < 0.0001), although the effect was less pronounced than that observed for the platinum(IV) prodrugs. FCCP, the positive control, produced the greatest reduction in MtMP from 24 and 48 h (*p* < 0.001), which remained significant at 72 h (*p* < 0.0001).

### 2.9 Morphological Changes in Microtubule Organization

**FIGURE 9:**
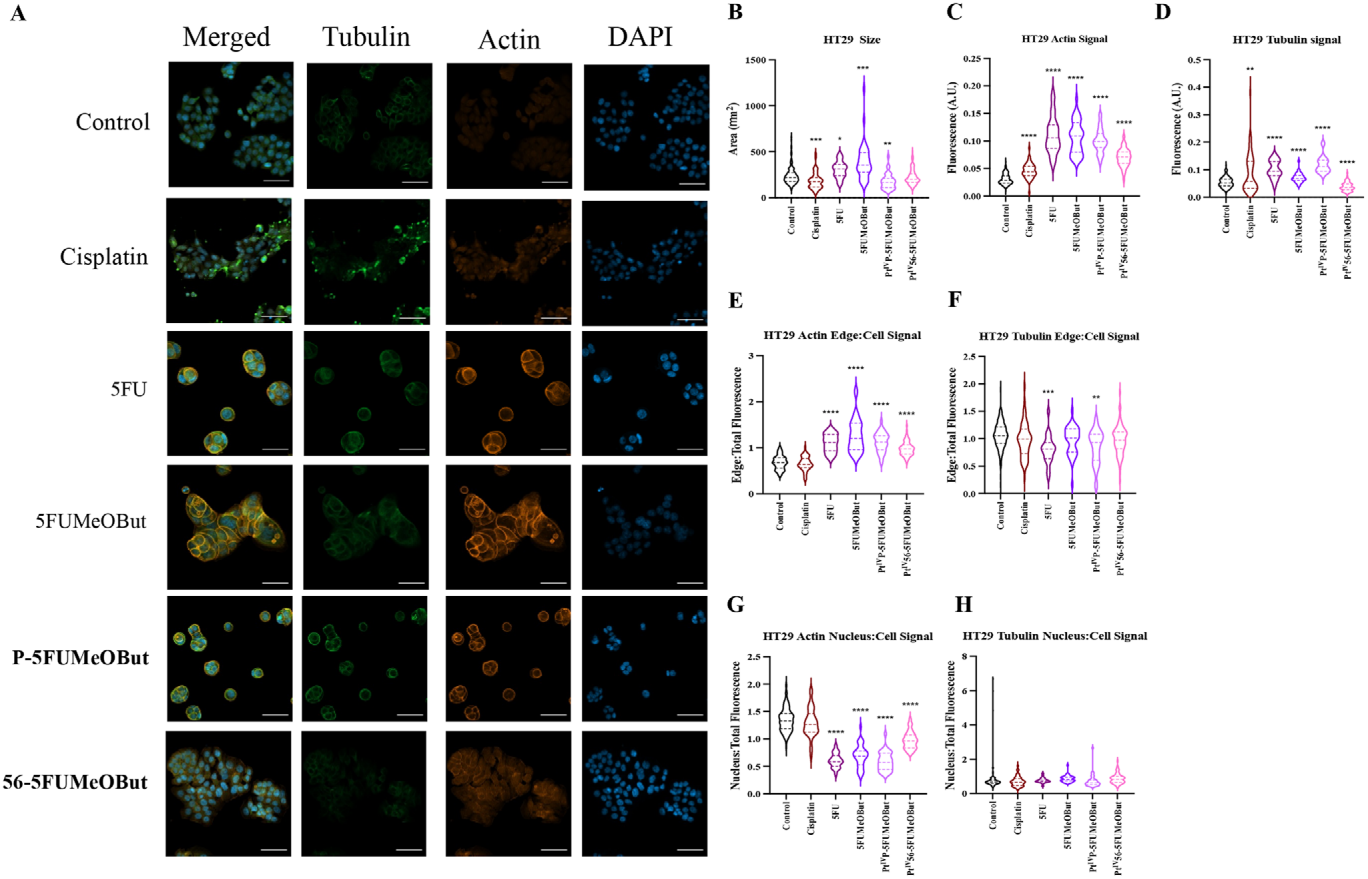
Effect of fluorouracil‐platinum(IV) prodrugs, 5FU, 5FUMeOBut or cisplatin on β‐tubulin and F‐actin in HT29 cells were assessed by immunofluorescence at 72 h post‐treatment, as detailed in Method 5.12. (A). Airy scan images of HT29 cells at 20 ×. (B). Cell size (μ m^2^) of HT29 cells. (C). Actin expression levels. (D). Tubulin expression levels. (E). Edge‐to‐cell ratio of actin. (F). Edge‐to‐cell ratio of tubulin. (G). Nucleus‐to‐cell ratio of actin. (H). Nucleus‐to‐cell ratio of tubulin. The data for cisplatin was obtained from previously reported data run in parallel [44]. Data points denote mean ± SEM of *n* = 3 from three separate experiments. * *p* < 0.05, ** *p* < 0.01, *** *p* < 0.001, and **** *p* < 0.0001, as measured by one‐way ANOVA.

HT29 cells were examined by confocal microscopy, for changes in microtubule intensity and architecture after 72 h of treatment with, **Pt^IV^P‐5FUMeOBut, Pt^IV^56‐5FUMeOBut**, 5FU, 5FUMeOBut or cisplatin (Figures 9 and S8). Cell size, tubulin intensity, and actin intensity were quantified. Furthermore, the fluorescence signal of actin or tubulin at the edge of the cell and in the nucleus were measured. Phalloidin staining of F‐actin revealed a distinct aggregation of actin filaments at the cytosol following treatment with **Pt^IV^P‐5FUMeOBut**, **Pt^IV^56‐5FUMeOBut**, 5FU or 5FUMeOBut (Figure S8). This translates to the actin total cell expression (Figure 9A, C) with significant increase (*p* < 0.0001) for all treatments, primarily observed at the edge of the cell (Figure 9E). While actin nuclear expression was decreased across all treatments except cisplatin, which suggest a different mechanism of action (Figure 9G).

The observed changes in actin are characteristic of cells undergoing apoptosis induced cell death [70, 71, 72]. The total cellular expression of tubulin was significantly increased in cells treated with cisplatin (*p* < 0.01), 5FU (*p* < 0.0001), 5FUMeOBut (*p* < 0.0001), and **Pt^IV^P‐5FUMeOBut** (*p* < 0.0001). In contrast, expression was notably reduced in cells treated with **Pt^IV^56‐5FUMeOBut** (*p* < 0.0001), as shown in Figure 9A, D. A similar observation was previously reported for the prodrug, [Pt^IV^(5,6‐dimethyl‐1,10‐phenanthroline)(S,S‐DACH)(Chlorambucil)(OH)](NO_3_)_2_ (**Pt^IV^56CLB)** in HT29 treated cells [69]. The tubulin signal at the edge of the cell decreased for 5FU (*p* < 0.001) and **Pt^IV^P‐5FUMeOBut** (*p* < 0.01) compared to control (Figure 9F), while no significant changes were detected in the nuclear to cytosolic ratio of tubulin in treated cells (Figure 9H). Additionally, the HT29‐treated cells displayed morphological alterations in the cytoskeleton, in contrast to the normal filamentous distribution of actin and tubulin observed in the untreated (control) cells (Figures 9A and S8). A significant reduction in cell size was observed across the cells treated with **Pt^IV^P‐5FUMeOBut** (*p* < 0.01) or cisplatin (*p* < 0.001), while that of 5FU (*p* < 0.05) or 5FUMeOBut (*p* < 0.001) increased in cell size (Figure 9B).

### 2.10 Wound Healing Assay

**FIGURE 10:**
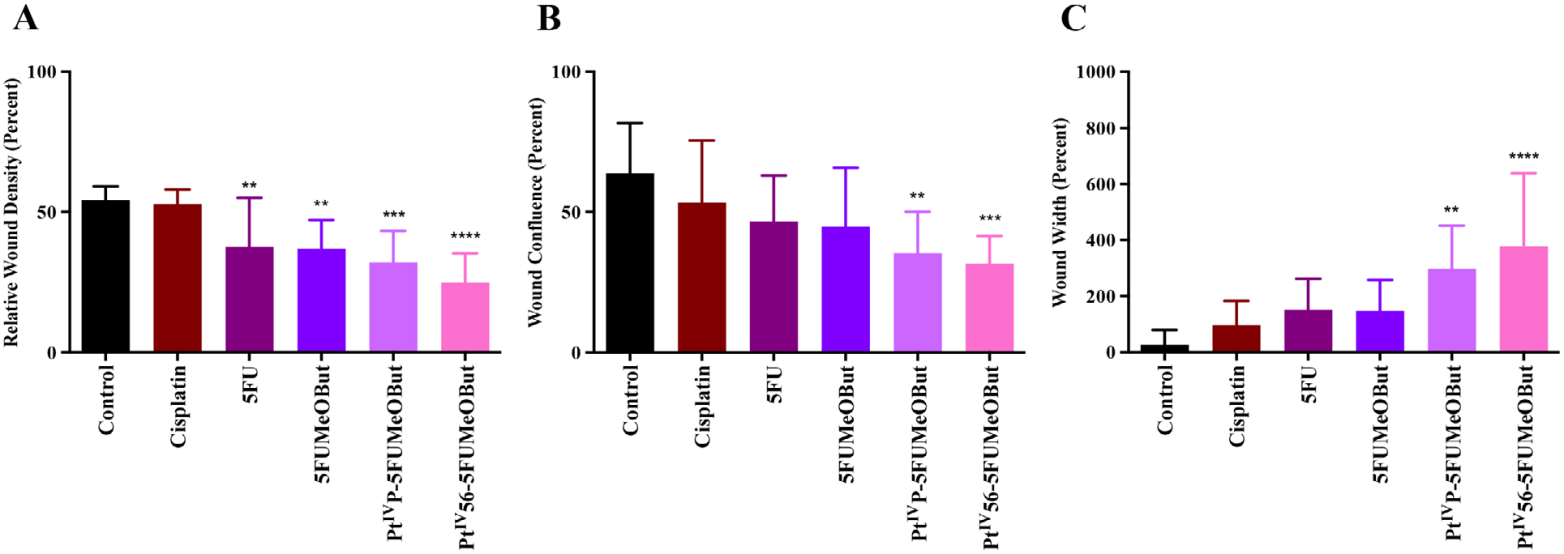
Cell wound closure. (A). Percent relative wound density, (B). wound confluence and (C). wound width measured upon treatment with **Pt^IV^P‐5FUMeOBut** or **Pt^IV^56‐5FUMeOBut** prodrugs, as well as 5FU, 5FUMeOBut or cisplatin in HT29 cells up to 72 h as described in Method 5.13. Cisplatin data was obtained from previously reported data run in parallel [44]. Data points denote mean ± SEM of *n* = 3 from three separate experiments where samples were run in triplicate. * *p* < 0.05, ** *p* < 0.01, *** *p* < 0.001, and **** *p* < 0.0001 compared with control, as measured by one‐way ANOVA.

Tumor metastasis occurs when cancer cells invade adjacent tissues and blood vessels, enabling them to migrate in response to chemical signals and adhere to distant sites [73, 74]. A 2D monolayer of HT29 cells was wounded and examined for 72 h after treatment with prodrugs, **Pt^IV^P‐5FUMeOBut** or **Pt^IV^56‐5FUMeOBut**, 5FU, 5FUMeOBut or cisplatin (Figures 10, S9 and S10). The resulting percent relative wound density, wound confluence and wound width were measured (Figure 10 and S9). HT29 cells treated with **Pt^IV^P‐5FUMeOBut** exhibited significantly reduced wound density (*p* < 0.001) and confluence (*p* < 0.01) (Figure 10A, B), which was measured for **Pt^IV^56‐5FUMeOBut** (*p* < 0.0001). An increase in wound width was measured across treatments with a notable increase for **Pt^IV^P‐5FUMeOBut** (*p* < 0.01) or **Pt^IV^56‐5FUMeOBut** (*p* < 0.0001) treated cells (Figure 10C). Compared to cisplatin, the relative wound density was significantly decreased for **Pt^IV^P‐5FUMeOBut** (*p* < 0.001) and **Pt^IV^56‐5FUMeOBut** (*p* < 0.0001). The wound width measured for **Pt^IV^P‐5FUMeOBut** and **Pt^IV^56‐5FUMeOBut** remained significantly higher compared to cisplatin with *p* < 0.05 and *p* < 0.001, respectively.

### 2.11 Mechanism of Cell Death

**FIGURE 11:**
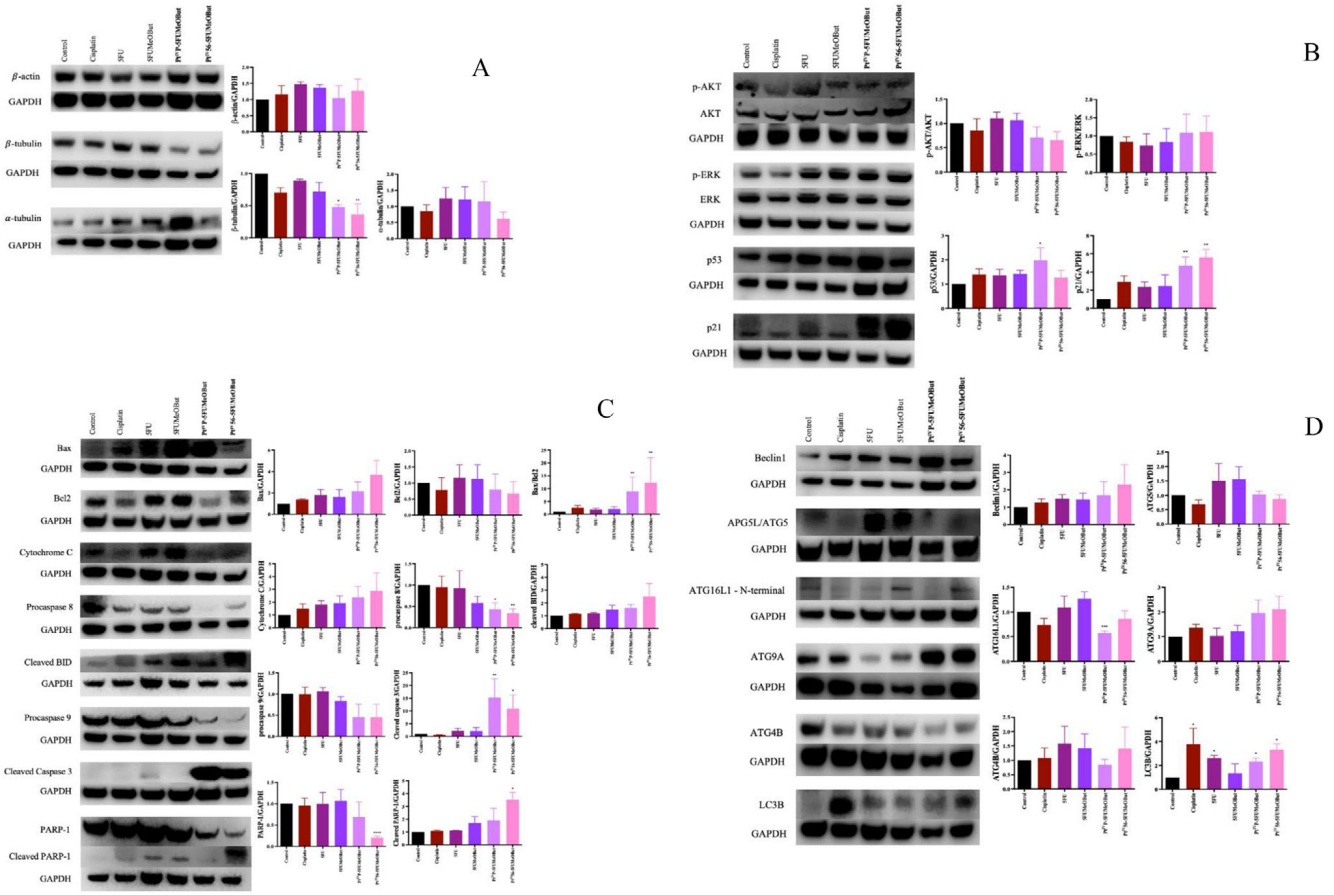
Protein expression in HT29 cells following 72 h treatment with **Pt^IV^P‐5FUMeOBut**, **Pt^IV^56‐5FUMeOBut**, 5FU, 5FUMeOBut, or cisplatin as outlined in Method 5.14. (A). Markers of the microtubule cytoskeleton. (B). Cell proliferation markers. (C). Markers of intrinsic and extrinsic apoptosis. (D). Autophagy markers. Details of the antibodies used, including molecular weights, are provided in Table S6. Data points denote mean ± SEM. *n* = 3 from three independent experiments. * *p* < 0.05, ** *p* < 0.01, *** *p* < 0.001, and **** *p* < 0.0001 compared with control, as measured by unpaired Student's t‐test.

The protein expression changes in microtubule cytoskeleton, cell proliferation, key apoptotic and autophagic proteins in HT29 cells were measured 72 h after treatment with **Pt^IV^P‐5FUMeOBut**, **Pt^IV^56‐5FUMeOBut**, 5FU, 5FUMeOBut or cisplatin (Figure 11). Results show some alterations to the microtubule cytoskeleton markers with a notable decrease in β‐tubulin in **Pt^IV^5FUMeOBut** (*p* < 0.05) and **Pt^IV^56‐5FUMeOBut** (*p* < 0.01), treatments which is accompanied by an observed decrease in α‐tubulin for **Pt^IV^56‐5FUMeOBut** treatment (Figure 11A).

Prodrug treatment also influenced the expression of cellular proliferation markers (Figure 11B). A decrease in p‐AKT/AKT for **Pt^IV^P‐5FUMeOBut** and **Pt^IV^56‐5FUMeOBut** treatments was observed but with no changes in p‐ERK/ERK across treatments. An increase in p53 was detected for all treatments with significance for **Pt^IV^P‐5FUMeOBut** (*p* < 0.05) whereas p21 exhibited an increase for all treatments in both prodrug treatments: **Pt^IV^P‐5FUMeOBut** (*p* < 0.01) and in **Pt^IV^56‐5FUMeOBut** (*p* < 0.01).

The mitochondrial intrinsic and extrinsic mechanism of cell death was also investigated (Figure 11C). The pro‐apoptotic marker BAX significantly increased in all treatment groups, while the expression of the anti‐apoptotic marker Bcl2 was notably decreased in cells treated with **Pt^IV^P‐5FUMeOBut** or **Pt^IV^56‐5FUMeOBut**. A significant increase in Bax/Bcl2 was exhibited for **Pt^IV^P‐5FUMeOBut** (*p* < 0.01) or **Pt^IV^56‐5FUMeOBut** (*p* < 0.01) treated cells signifying a pro‐apoptotic mechanism. Cytochrome c was significantly elevated in all treatments. Procaspase 8 was significantly reduced for **Pt^IV^P‐5FUMeOBut** (*p* < 0.05) and **Pt^IV^56‐5FUMeOBut** (*p* < 0.01) together with increased cleaved BID in both prodrugs indicating the extrinsic pathway is activated. The executioner caspases, caspase 9 and 3, were also activated, as shown by a decrease in procaspase 9 expression across both prodrugs and a significant increase in cleaved caspase 3 in cells treated with **Pt^IV^P‐5FUMeOBut** (*p* < 0.01) and **Pt^IV^56‐5FUMeOBut** (*p* < 0.05). The levels of PARP‐1 were considerably reduced for **Pt^IV^56‐5FUMeOBut** (*p* < 0.0001), with an increase in cleaved PARP‐1 in **Pt^IV^56‐5FUMeOBut** (*p* < 0.05) treated cells.

Markers of autophagy were also investigated in HT29 treated cells (Figure 11D). Beclin‐1 showed an increase for all treatments. The expression levels of ATG5 and ATG16L1 were found to increase with ligands 5FU and 5FUMeOBut treatments, while a significant decrease was exhibited by **Pt^IV^P‐5FUMeOBut** (*p* < 0.001). ATG9A showed an increase expression for prodrug treatments. ATG4B increased in 5FU, 5FUMeOBut and **Pt^IV^56‐5FUMeOBut** treatments. The levels of LC3B were significantly elevated with cisplatin, 5FU, **Pt^IV^P‐5FUMeOBut** and **Pt^IV^56‐5FUMeOBut** (*p* < 0.05).

### 2.12 Proteomics

**FIGURE 12:**
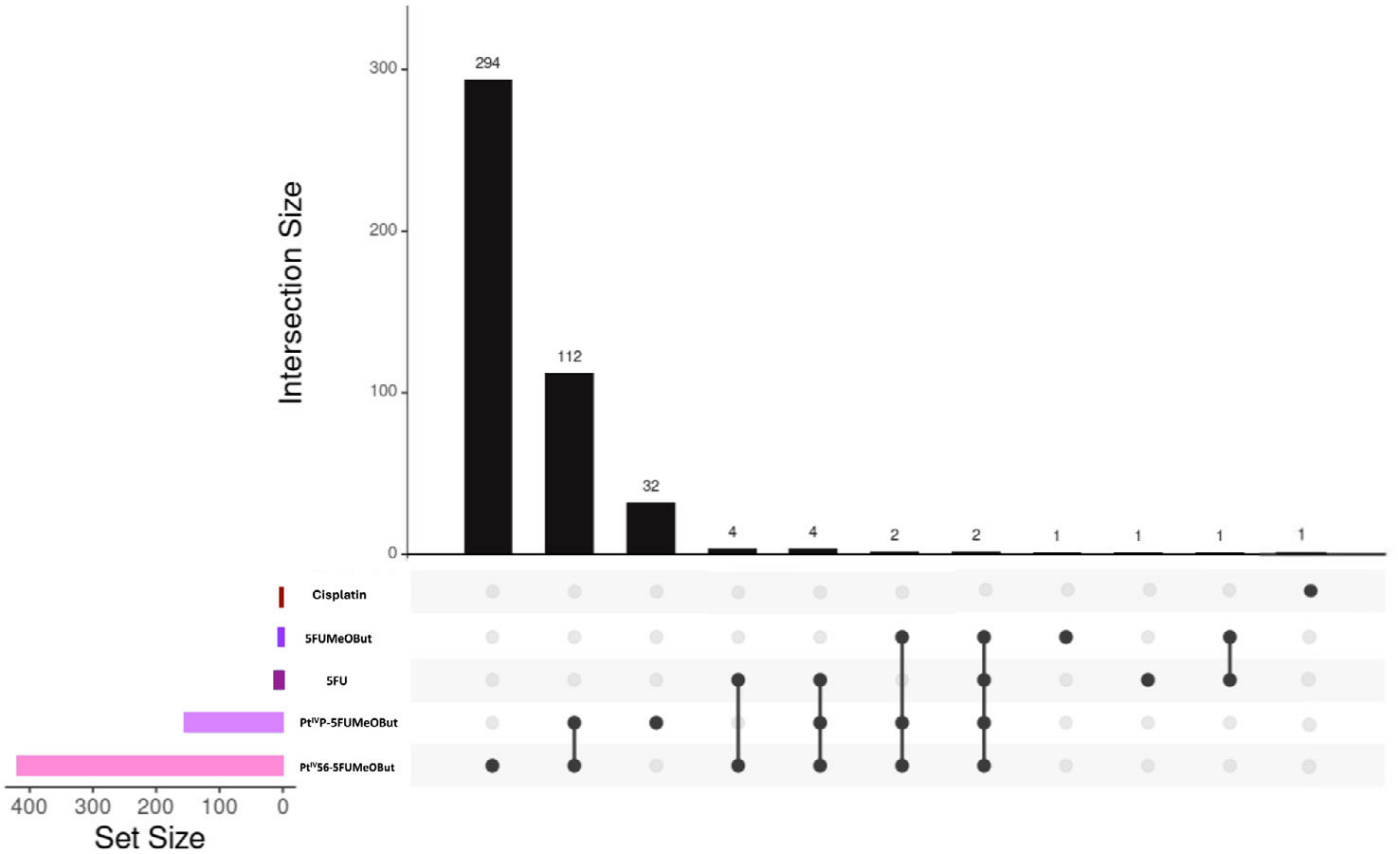
Differential proteins upon treatment with **Pt^IV^P‐5FUMeOBut**, **Pt^IV^56‐5FUMeOBut** prodrugs, 5FU, 5FUMeOBut, and cisplatin in HT29 cells at 72 h. UpSet plot summarizes the differential protein expression analysis of prodrugs and ligands. The total number of proteins exhibiting variations in log2‐fold change expression for each complex is shown in the horizontal bar graph at the bottom left of each image. The joined black circles to the right of the bar graphs indicate shared differentially expressed proteins across the complex comparisons on the left. The vertical bar graph at the top quantifies the number of proteins with similar log2‐fold change expression differences across the drug comparisons.

**FIGURE 13:**
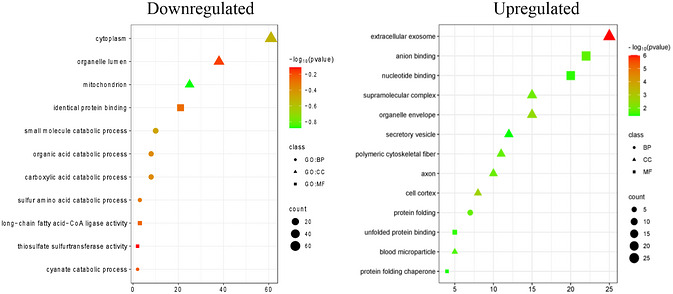
Proteomic analysis of HT29 upon treatment with Pt^IV^P‐5FUMeOBut. GO enriched biological processes, cellular components, and molecular function in HT29, downregulated and upregulated proteins. Data points denote mean ± SEM. *n* = 3 from three separate experiments.

**FIGURE 14:**
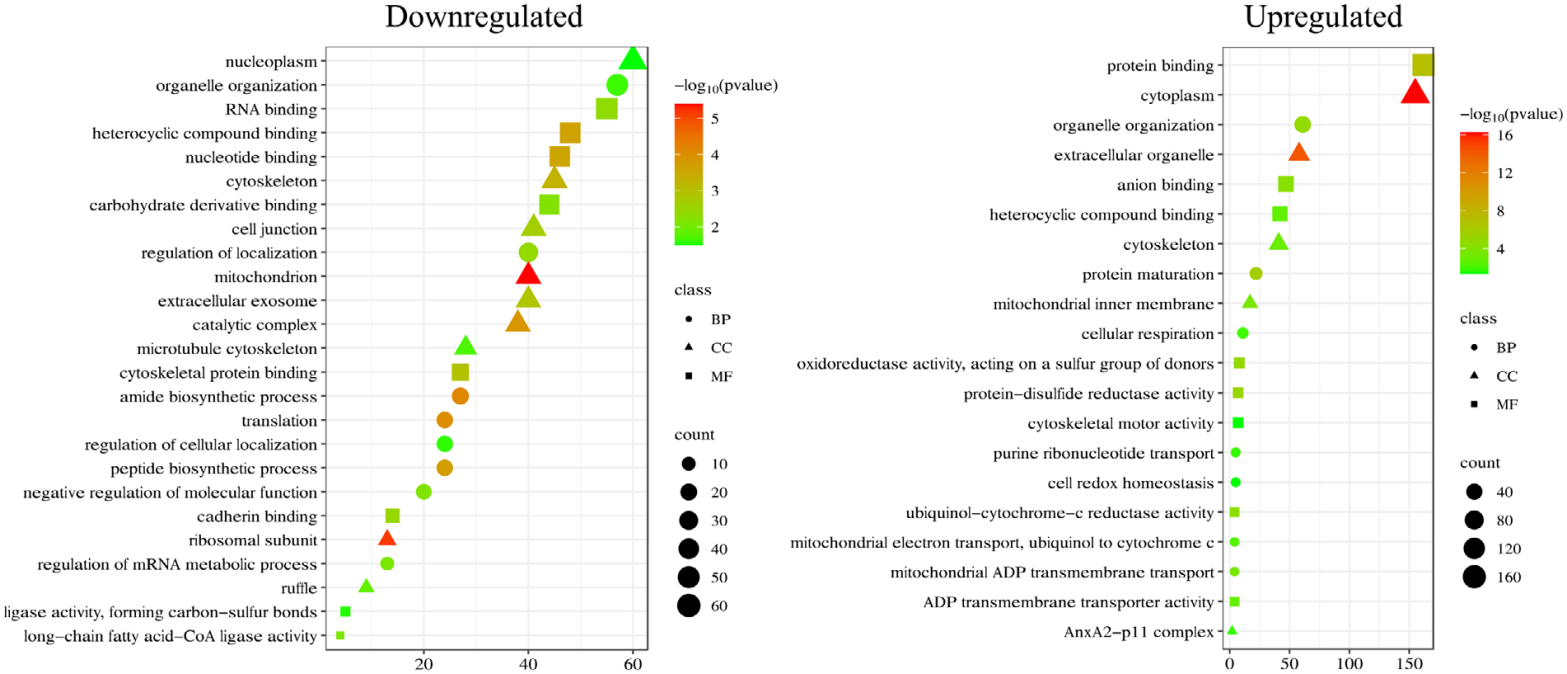
Proteomic analysis of HT29 upon treatment with Pt^IV^56‐5FUMeOBut. GO enriched biological processes, cellular components, and molecular function in HT29, downregulated and upregulated proteins. Data points denote mean ± SEM. *n* = 3 from three separate experiments.

The global proteome profile of HT29 cells treated with IC_50_ value concentrations for each investigated agent was determined at 72 h using quantitative proteomic techniques (Figures 12, 13, 14). A total of 1,859 HT29 proteins were identified across all paired samples that passed quality control in all three replicates for each drug treatment. PCA plots in Figures S11–15A show the total variances in proteome expression profiles for treated samples compared to control.

The number of up‐ and downregulated proteins are shown across all the treatments (Student's unpaired t‐test, *p* < 0.05, fold change > 2) (Figures S11–15B). Of all the investigated agents, **Pt^IV^P‐5FUMeOBut** and **Pt^IV^56‐5FUMeOBut**, showed dysregulated proteins post‐treatment correlating with the potency of the prodrugs. The ligand 5FU (Figure S11), 5FUMeOBut (Figure S12), and cisplatin (Figure S13) did not exhibit a similar effect after 72 h of treatment. HT29 cells treated with cisplatin did not exhibit many DEPs, TK1 was upregulated and DYNLL1 downregulated (Figure S13C). While **Pt^IV^P‐5FUMeOBut** (Figure S14C) and **Pt^IV^56‐5FUMeOBut** (Figure S15C), exhibited several dysregulated proteins, with PDP2 increased expression and ACFS2 decreased expression a common observation between the two treatments. The number of dysregulated proteins affected by treatment from highest to lowest were **Pt^IV^56‐5FUMeOBut** (Figure S15B) > **Pt^IV^P‐5FUMeOBut** (Figure S14B) > 5FUMeOBut (Figure S12B) > 5FU (Figure S11B) > cisplatin (Figure S13B). DEPs identified as intersecting for all treatments in HT29 are shown in an UpSet plot (Figure 12). There were 112 DEPs shared to both prodrugs. **Pt^IV^56‐5FUMeOBut** treatment had the most DEPs with 294> **Pt^IV^P‐5FUMeOBut** with 32 DEPs > 5FU, 5FUMeOBut and cisplatin with 1 DEP when used alone.

Based on GO annotations, the functional ontology enrichment analysis of drug‐specific up‐ and down‐regulated proteins were studied. Figures 13 and 14 present the findings from the three GO biological process, molecular function, and cellular component. GO analysis presented the dysregulated pathways for **Pt^IV^P‐5FUMeOBut** treated HT29 cells were linked to, metabolic processes, mitochondrion, protein folding, and organelle enveloping (Figures 13), while that for **Pt^IV^56‐5FUMeOBut** treated in HT29 cells were associated with cytoskeletal organization, organelle organization, RNA binding, catalytic activity, mitochondrial inner membrane, mitochondrial transmembrane transport, biosynthetic processes, and translation (Figure 14).

## 3 Discussion

The fluorouracil‐platinum(IV) prodrugs were studied to explore their cellular mechanisms of action and assess their therapeutic potential. These prodrugs have previously demonstrated broad antineoplastic activity across multiple cancer cell lines including colorectal, breast, brain, lung and skin cancers [39]. The present study extends these findings by providing a comprehensive cellular and molecular evaluation of their cytotoxic effects, guiding their evaluation in future in vivo investigations.

Cytotoxicity studies confirmed that both **Pt^IV^P‐5FUMeOBut** and **Pt^IV^56‐5FUMeOBut** retain superior anticancer activity relative to cisplatin and the free ligands with **Pt^IV^56‐5FUMeOBut** consistently exhibiting the highest potency (Table 1) [39]. Platinum(IV) complexes have the potential to avoid the typical harmful side effects associated with platinum(II) complexes by selectively reducing and activating within the hypoxic and low‐pH environments of cancer cells [32, 75, 76, 77]. Assessment in nontumorigenic MCF10A cells demonstrated improved selectivity for cancer cells (Tables 1 and 2), supporting the premised that the prodrugs can preferentially activate within the hypoxic and reductive tumor microenvironment. The SCI was higher for **Pt^IV^P‐5FUMeOBut** and **Pt^IV^56‐5FUMeOBut** despite the IC_50_ value being in the low micromolar range, which implies the agents have a better selectivity for cancer cells, as demonstrated by their greater SCIs ranging from 5–33 compared to cisplatin, 5FU and 5FUMeOBut which ranged from 0–18. Although reduced cytotoxicity was observed in 3D spheroids compared to 2D monolayers, the prodrugs remained substantially more potent than cisplatin, with over a 51‐fold greater potency (Table 1), highlighting their potential efficacy under physiologically relevant conditions that better mimic tumor organization in vivo.

At an incubation concentration of 3 µM, both fluorouracil‐platinum(IV) prodrugs (Figures 2,S4, and Table S3) showed similar cellular accumulation to the platinum(II) precursors and greater than that of the platinum(IV) scaffolds [44]. **Pt^IV^56‐5FUMeOBut** reached the highest intracellular concentration, likely contributing to its superior cytotoxicity relative to cisplatin. Uptake of both prodrugs by HT29 cells was time‐dependent, accounting for the continuous increase over time without reaching a plateau. This can be explained by their mechanism of action, as reductive activation within the cell is required for cytotoxicity observed at 72 h [24]. The high intracellular/extracellular ratio suggests that these prodrugs utilize an active transport mechanism (Table S3).

Platinum(IV) prodrugs are likely taken up by cells through multiple mechanisms, including passive or facilitated diffusion, endocytosis, and active transport mediated by ATP‐dependent transport proteins [78, 79]. After blocking the relevant mechanism and comparing the results at ideal conditions at 37°C, the transport mechanism was measured by Pt uptake *via* ICP‐MS. By blocking active transport mechanisms at 4°C, the impact on active transport was investigated [80]. **Pt^IV^P‐5FUMeOBut** and **Pt^IV^56‐5FUMeOBut** showed a significant reduction in uptake at 4°C, indicating their use of this mechanism for cellular drug uptake.

Clathrin‐mediated endocytosis and SLC7A5 receptor contributed significantly to the uptake of **Pt^IV^P‐5FUMeOBut** (*p* < 0.05), similar to cisplatin (*p* < 0.01) [81, 82, 83, 84], whereas **Pt^IV^56‐5FUMeOBut** showed a decrease upon inhibition. SLC7A5 is an amino acid transporter overexpressed in many cancer cells, responsible for importing large neutral amino acids critical for tumor growth and survival. Inhibiting this transporter allowed us to assess whether the prodrugs exploit this pathway for cellular entry. The observed difference between the two prodrugs suggests that **Pt^IV^P‐5FUMeOBut** relies more on SLC7A5‐mediated transport than **Pt^IV^56‐5FUMeOBut**. The primary protein carrier of iron; transferrin; overexpressed in cancer cells, was also studied for the impact on mechanism of uptake by the fluorouracil‐platinum(IV) prodrugs. While TfR inhibition showed no significant reduction, which is consistent with previous reports for the platinum(II) precursors [44], a decrease was observed for **Pt^IV^P‐5FUMeOBut** and **Pt^IV^56‐5FUMeOBut**. This study suggests the fluorouracil‐platinum(IV) prodrugs may utilize many intracellular pathways, with active transport acting as the primary mechanism of cell uptake (Figure 3).

Subcellular fractionation revealed distinct intracellular distributions for the fluorouracil‐platinum(IV) prodrugs(Figure 4). **Pt^IV^56‐5FUMeOBut** accumulated predominately in the cytoskeletal fraction, consistent with prior observations for the platinum(II) precursor, **Pt^II^56ME*S*** [44], whereas **Pt^IV^P‐5FUMeOBut** showed the highest concentrations at the membrane/particulate fraction, followed by cytoskeletal and nuclear fractions, mirroring the pattern of **Pt^II^PHEN*SS*** in HT29 cells [44]. Interestingly, the chlorambucil‐platinum(IV) prodrug was only significantly localized to the cytoskeletal fraction [69], highlighting that axial ligand identity can markedly influence intracellular localization. These differences suggest that interactions with cytoskeleton, cellular membranes, or nuclear DNA, particularly *via* the intercalating ligand, 5FU may underlie the distinct mechanisms of action of each prodrug, contributing to cytoskeletal disruption, interference with biosynthetic processes, and the induction of apoptosis [44, 85, 86, 87].

Cell death analysis showed that the fluorouracil‐platinum(IV) prodrugs primarily induce programmed cell death through apoptosis (*p* < 0.0001) (Figure 5). Increased phosphatidylserine to the outer leaflet of the cell membrane when the cell is in early and late stages of apoptosis is detected by Annexin‐V [88, 89]. Cells in the late apoptosis and necrosis stage lose their membrane integrity, which increases their permeability to the DNA‐binding PI stain [90]. Cell cycle analysis was used to provide mechanistic context for drug‐induced cell death; drugs that kill cancer cells that are dividing, or cell‐cycle non‐specific; drugs that kill cancer cells when they are resting [91, 92]. The redistribution of cells from G0/G1 into S phase observed across all treatments is consistent with preferential targeting of proliferating cells. The additional increase in G2+M for **Pt^IV^56‐5FUMeOBut** (Figure 6) suggests a distinct effect on cell‐cycle progression, which may relate to its enhanced localization within the cytoskeletal fraction, as agents that disrupt microtubule dynamics are known to induce G2+M arrest [93, 94].

The production of ROS was investigated (Figure 7) and found to be significantly higher than the control at 48 and 72 h across all agents. This indicates that intracellular stress induced by the investigated agents causes damage to cellular components such as proteins, lipids, and nucleic acids, ultimately prompting apoptosis [24, 44, 95]. Accordingly the MtMP was studied to confirm the effect of ROS on mitochondrial hypopolarization as has been observed previously by platinum(II) precursors [44]. Cisplatin has been reported to produce ROS and subsequently effect MtMP [49, 96, 97]. Mitochondria is essential for cellular energy and for normal cell function [98]. Chemotherapeutic agents have been reported to destroy the MtMP that induces the activation of the intrinsic pathway of apoptosis in the mitochondria [99]. Platinum(II) precursors and platinum(IV) scaffolds for the prodrugs were shown to cause a significant decrease in MtMP in HT29 cells [44]. In line with these reports, both fluorouracil‐platinum(IV) prodrugs, as well as 5FU and 5FUMeOBut significantly reduced MtMP in HT29 cells at 24, 48, and 72 h (Figure 8) supporting a ROS‐associated mechanism of mitochondrial hypopolarization contributing to cell death.

Taken together, the enhanced ROS production observed for the prodrugs relative to the platinum(II) precursors suggests that the released 5FU contributes functionally to the overall cellular response. Elevated ROS levels may also have implications for DNA biosynthesis pathways relevant to the released 5FU. Oxidative stress is known to induce DNA lesions and perturb nucleotide pools, increasing cellular reliance on de novo thymidylate synthesis for DNA repair and replication. Under such conditions, inhibition of thymidylate synthase by intracellularly liberated 5FU would be expected to exacerbate DNA biosynthetic stress and limit the repair of ROS‐mediated damage, thereby amplifying cytotoxicity beyond that produced by the platinum scaffold alone [4, 9, 39, 45, 46, 47].

Importantly, the objective of the 5FU‐platinum(IV) prodrug design is not to recreate the effects of a classical co‐administration experiment, but to achieve coordinated intracellular delivery and release of two bioactive components with distinct mechanisms of action. While free 5FU does not reach its standalone IC_50_ at concentrations corresponding to platinum(II)‐driven cytotoxicity, this does not preclude a functional contribution following intracellular release. In this context, sub‐cytotoxic levels of 5FU may sensitize cells to platinum‐induced and ROS‐mediated stress by impairing nucleotide metabolism and restricting DNA repair capacity. Consistent with this concept, the 5FU‐platinum(IV) prodrugs induced higher intracellular ROS levels than their corresponding platinum(II) complexes alone (Table S4), indicating a cooperative intracellular effect.

Previous studies have shown that platinum(II) precursors and platinum(IV) dihydroxy scaffolds can disrupt actin and tubulin organization in cancer cells [44, 50]. In this context, the effects of **Pt^IV^P‐5FUMeOBut** and **Pt^IV^56‐5FUMeOBut** on cytoskeletal components (Figure 9) were examined to determine whether the prodrugs elicited responses comparable to their platinum(II) precursors. Alterations in actin and tubulin organization were associated with changes in cell morphology consistent with apoptotic progression, including reduced cell size following **Pt^IV^P‐5FUMeOBut** and cisplatin treatment [100]. In contrast, increased cell size observed with 5FU and 5FUMeOBut treatment is indicative of necrotic cell death characterized by cell swelling [101]. Actin remodeling plays a central role in apoptosis through its involvement in DNA fragmentation and membrane blebbing mediated by caspase‐3 activation [44, 72, 102]. Consistent with this mechanism, phalloidin staining revealed actin filament aggregation at the cell periphery, resulting in an overall increase in actin expression for all investigated agents (Figures 9C and E). Microtubules, which are essential for mitosis, intracellular transport, and cell motility, represent an established chemotherapeutic target due to their role in mitotic spindle formation and cell‐cycle progression [103, 104, 105]. Altered tubulin organization following treatment, characterized by aggregation at the cell periphery, supports disruption of microtubule architecture. Notably, tubulin expression was reduced in **Pt^IV^56‐5FUMeOBut** treated cells, consistent with previous observations for **Pt^IV^56CLB** [69]. This suggests a shared mechanism linked to the platinum(II) precursor; **Pt^II^56ME*SS***, which has been reported to alter microtubule dynamics and induce G2+M cell cycle arrest [49, 50, 106, 107, 108].

Tumor metastasis is initiated by directed cell migration and adhesion to the ECM, processes driven by membrane protrusion and cytoskeletal remodeling [109, 110, 111]. In this context, the impaired wound closure observed following treatment with **Pt^IV^P‐5FUMeOBut** and **Pt^IV^56‐5FUMeOBut** (Figures 10, S9 and S10) indicates a reduction in migratory and adhesive capacity. The sustained wound width, together with decreased cell density and confluency, is consistent with a reduced tendency of cells to spread and migrate.

We note that Western blot analyses were performed at 72 h at IC_50_ concentrations, primarily reflecting the surviving fraction of HT29 cells. While this may include adaptive or stress‐response mechanisms, these data provide biologically meaningful insights into the cellular pathways engaged by the prodrugs. Importantly, the fluorouracil‐platinum(IV) prodrugs consistently modulated apoptotic, autophagic, and cytoskeletal pathways, indicating their potential to engage complementary cell death processes and overcome resistance mechanisms. Microtubule‐associated proteins were examined to assess cytoskeletal effects. Significant reduction in β‐tubulin, but not β‐actin, indicates that the prodrugs disrupt microtubule dynamics, consistent with previous observations for **Pt^II^56ME*SS*** in MDA‐MB‐231 cells [44, 50]. Tumor suppressor proteins p53 and p21 were evaluated (Figure 11B). HT29 cells carry mutant p53, so observed increases likely reflect adaptive stress responses in the surviving population rather than canonical p53‐mediated G1/S checkpoint activation. Nonetheless, p21 upregulation suggests that p53‐independent mechanisms may contribute to modulation of cell proliferation [112, 113]. Signaling pathways involved in survival and proliferation, including PI3K‐AKT and Ras‐ERK, have been linked to cellular invasion, migration, and survival [114, 115]. ROS generation associated with DNA damage can activate p53 and p21, processes that are regulated by ERK [116, 117]. In our study, p‐AKT/AKT was reduced by **Pt^IV^P‐5FUMeOBut** and **Pt^IV^56‐5FUMeOBut**, consistent with induction of apoptosis, whereas minor increases in pERK likely reflect heterogeneous responses in the surviving cell fraction, in line with quantification results. AKT regulates apoptosis through phosphorylation of BAX at Ser184 and BAD at Ser136, preventing mitochondrial cytochrome c release and lowering caspase 9 activity, with MDM2 suppression further modulating p53 function [118, 119, 120]. Effectors of apoptosis are closely linked to PI3K‐AKT and Ras‐ERK signaling [121, 122, 123]. The balance of Bcl2 family proteins, particularly Bax and Bcl2, governs mitochondrial‐mediated apoptosis, with Bax promoting cytochrome c release and Bcl2 inhibiting it [101, 124, 125]. The observed increase in the Bax/Bcl2 ratio and cytochrome c release in prodrug‐treated cells indicates effective engagement of the intrinsic apoptotic pathway. The extrinsic pathway, mediated by death receptors and caspase 8 activation, converges on mitochondria through Bid cleavage, amplifying apoptosis [126]. These pathways share downstream execution markers, including caspase 9 and 3 and PARP‐1, which coordinate DNA fragmentation and cell death [124, 127, 128, 129]. Overall, these observations suggest that fluorouracil‐platinum(IV) prodrugs robustly activate both intrinsic and extrinsic apoptotic mechanisms in HT29 cells. Autophagy markers, including LC3B, Beclin‐1, and ATG proteins, were also modulated, highlighting multimechanistic cell death potential. We note that the 72 h time point reflects the surviving cell fraction; nevertheless, these measurements provide a meaningful overview of apoptotic, autophagic, and cytoskeletal responses, with all quantifications normalized to respective controls. The suppression of autophagy associated cell death is linked to the Bcl2 complex and Beclin‐1, a key marker of autophagy. Downregulation of Bcl2 opens a crosstalk between autophagy and apoptosis [130]. The suppression of PI3K signalling pathways suggests mTOR may have been suppressed, activating autophagy markers [131, 132]. Autophagy‐related proteins (ATGs) operate through four main systems: the class III PI3K complex (e.g., ATG14, Beclin‐1), the ATG12 conjugation system (e.g., ATG16L1, ATG5), the LC3 conjugation system (e.g., LC3, ATG4), and the ATG9 trafficking system (e.g., ATG9A) [133, 134, 135]. Key markers such as ATG5, ATG9A, and LC3B coordinate vesicle formation, lipid trafficking, and autophagosome‐lysosome fusion, while interactions between ATG4B and Bcl2 facilitate crosstalk between autophagy and apoptosis [133, 134, 135, 136, 137, 138, 139, 140, 141, 142]. The observed modulation of these markers, including increases in Beclin‐1, ATG9A, and LC3B following prodrug treatment, indicates that fluorouracil‐platinum(IV) prodrugs engage autophagy alongside apoptosis. This multimechanistic activity may enhance cancer cell killing and mitigate resistance, consistent with the potential benefits of combining complementary mechanisms in a single prodrug entity [143, 144].

Proteome profiling revealed distinct drug‐specific effects, with **Pt^IV^56‐5FUMeOBut** emerging as the most potent prodrug, inducing the greatest dysregulation of proteins. **Pt^IV^P‐5FUMeOBut** showed broadly similar patterns, whereas the ligands, 5FU, 5FUMeOBut, and cisplatin had more limited effects [44, 69, 145]. GO and network analysis indicate that both prodrugs disrupt translation, mitochondrial function, organelle organization, biosynthetic processes, protein folding, and cytoskeletal dynamics, consistent with classical platinum drug activity [16, 146]. **Pt^IV^56‐5FUMeOBut** additionally enhanced cytochrome complex activity, mitochondrial ATP synthesis, electron transport, and oxidoreductase functions, suggesting ROS‐mediated DNA damage [147, 148]. Protein network analysis further highlighted dysregulation in mitochondrial transport, RNA binding, metabolism, and peptide biosynthesis, with **Pt^IV^56‐5FUMeOBut** producing the highest number of DEPs (294), surpassing **Pt^IV^56CLB** (149). These findings support its classification as a highly potent, multimechanistic chemotherapy agent.

HT29 cancer cells and spheroids responded effectively to fluorouracil‐platinum(IV) prodrugs, which were primarily taken up via active transport, with **Pt^IV^56‐5FUMeOBut** showing predominant cytoskeletal accumulation. These prodrugs induced ROS formation, decreased MtMP, and altered the microtubule organization, triggering apoptosis through both extrinsic and intrinsic signaling pathways. Autophagy‐dependent cell death may also contribute their overall cytotoxic effect. Collectively, these finding highlight the multimechanistic potential of **Pt^IV^P‐5FUMeOBut** and **Pt^IV^56‐5FUMeOBut** supporting their promise as effective and potentially safer treatment options for colorectal cancer.

## 4 Conclusions

Fluorouracil‐platinum(IV) prodrugs were effective against HT29 cancer cells, with active transport leading to a notable accumulation of the prodrugs in the cytoskeleton. These prodrugs decreased MtMP, increased ROS, and disrupted microtubule structure. The enhanced activity of the prodrugs relative to the platinum(II) precursors, particularly the greater ROS generation despite sub‐cytotoxic levels of released 5FU, supports a functional contribution of the fluorouracil component within the mutli‐action design. The complexes triggered apoptosis via both intrinsic and extrinsic apoptotic pathways, and may also engage autophagy‐dependent cell death. **Pt^IV^56‐5FUMeOBut**, in particular, showed strong multimechanistic activity, highlighting its potential as a safer and more effective alternative to cisplatin for colorectal cancer treatment. Future studies will focus on preclinical in vivo evaluation of these prodrugs to assess their efficacy, pharmacokinetics, and safety profile, as well as their potential in combination therapies, with the ultimate goal of advancing toward clinical application.

## 5 Experimental Section

### 5.1 Chemicals and Reagents

Cell culture media used included Dulbecco's Modified Eagle's Medium (DMEM) with 4.5 g/L glucose, L‐glutamine, sodium bicarbonate, and sodium pyruvate (Invitrogen #11995‐073), DMEM without phenol red (Invitrogen #21063‐029), and DMEM/F‐12 with HEPES (Invitrogen #11330‐057), all obtained from ThermoFisher Scientific (Brisbane, Australia) unless otherwise noted. Dulbecco's 1× Phosphate Buffered Saline (PBS) with MgCl_2_ and CaCl_2_ (Invitrogen #14040‐182), foetal bovine serum (FBS; Invitrogen #26140‐079), horse serum (HS; Invitrogen #16050‐122), 10× trypsin‐EDTA (Invitrogen #15400‐054), penicillin‐streptomycin (P/S; 5,000 U/mL; Invitrogen #15070‐063), and PageRuler Prestained Protein Ladder (Invitrogen #26617) were also sourced from ThermoFisher Scientific.

The MEGM Mammary Epithelial Cell Growth Medium SingleQuotsTM Kit (Lonza #CC‐4136) was purchased from Lonza (Sydney, Australia). Deionized water was generated using a MilliQ purification system (Millipore Australia, Sydney, Australia). All chemicals were of spectroscopic grade and used without further purification, with methanol obtained from Honeywell Research Chemicals (Morris Plains, USA). Additional reagents, including ammonium bicarbonate, dithiothreitol (DTT), chloroform, RNase A, Triton X‐100, NaCl, Tris base, bovine serum albumin (BSA), sucrose, and *D*‐phenylalanine, were purchased from Sigma–Aldrich (Sydney, Australia). The WST‐1 cell proliferation reagent was obtained from Roche Diagnostics (Indianapolis, USA). Concentrated buffers (10× TBS, 10× TGS, 10× TG, and 4× Laemmli sample buffer) were sourced from Bio‐Rad (Gladesville, Australia).

HRP‐linked secondary antibodies (anti‐rabbit #7074S, anti‐mouse #7076S) were purchased from Cell Signaling Technology (Danvers, USA), and primary antibodies were acquired from Abcam (Cambridge, USA), unless otherwise specified. Cell culture flasks and plates were obtained from Corning Inc. (Gilbert, USA), unless noted otherwise.

### 5.2 Cell Line and Complex Synthesis

Colorectal Cancer Cells (HT29) (ATCC, USA—HTB‐38) and non‐tumorigenic Immortalized Human Breast Epithelial Cells (MCF10A) (ATCC, USA—CRL‐10317) were purchased from the American Type Culture Collection. HT29 cells were cultured in DMEM supplemented with 10% Foetal Bovine Serum (FBS) and 1% penicillin–streptomycin in a humidified incubator at 37°C with 5% CO_2_. MCF10A were cultured in DMEM/F12 with 5% Horse Serum, 1% penicillin–streptomycin, and additional supplements including EGF (20 ng/mL), Hydrocortisone (0.5 mg/mL), Cholera Toxin (100 ng/mL), and Insulin (10 µg/mL) following MEGM kit formulation (excluding gentamicin/amphotericin) and in a humidified incubator at 37°C with 5% CO_2_. The synthesis and characterization of **5FUMeOBut**, **Pt^IV^P‐5FUMeOBut** and **Pt^IV^56‐5FUMeOBut** (Figure 1) followed previously reported methods, detailed in Supplementary Method S1 and Result S1 [149, 150, 151].

### 5.3 Cytotoxicity of Fluorouracil‐Platinum(IV) Prodrugs

Cells (HT29 or MCF10A, 1000 cells/well) were seeded in 96‐well flat bottom plates and incubated overnight. The platinum prodrugs, their bioactive ligand and cisplatin were evaluated for cytotoxicity using threefold dilutions ranging at 150 to 0.007 µM, following a previously described method [44]. Three separate experiments with triplicate samples were run. The SCI for **Pt^IV^P‐5FUMeOBut** and **Pt^IV^56‐5FUMeOBut**, ligands, and cisplatin were determined by dividing the IC_50_ (Table 1) values of the complexes or ligands in nontumorigenic MCF10A cells by those in HT29 cancer cells. The SCI reflects the therapeutic window; higher values indicate greater selectivity and efficacy against cancer cells [24, 152, 153].

### 5.4 Cytotoxicity Evaluation of Fluorouracil‐Platinum(IV) Prodrugs in a Cancer Spheroid Model

To assess the cytotoxicity of platinum prodrugs, 5FU, 5FUMeOBut, and cisplatin in a 3D cell culture model, HT29 cells were bioprinted using the noncontact drop‐on‐demand 3D RASTRUM bioprinter (Inventia Life Science, Alexandria, Australia) [51, 154]. The 3D large plug format with polyethylene glycol‐based bioink (Inventia, #Px02.09; see Table S1) was used. Following the automated fluid priming, 10,000 cells/well were printed into 96‐well tissue culture plates (Nunc MicroWell, Flat‐Bottom Microplate, ThermoFisher #167008), forming uniform 3D spheroids. Culture medium (DMEM) was added manually using multichannel pipettes, and spheroids were incubated for one week, as previously described [44]. Once spheroids had formed, media was refreshed (100 µL/well) and the platinum complexes, as well as bioactive ligands or cisplatin were added at concentrations ranging from 150 to 0.007 µM using threefold serial dilutions. Cytotoxicity was evaluated using the WST‐1 assay, following established protocol [44].

### 5.5 Cellular Uptake of Fluorouracil‐Platinum(IV) Prodrugs

HT29 cells (1 × 10^6^ cells/well) were seeded into 6‐well plates and incubated overnight to adhere. Cells were then treated with 3 µM of **Pt^IV^P‐5FUMeOBut** or **Pt^IV^56‐5FUMeOBut** for various time points (0, 0.5, 1, 3, 6, 12, 24, and 30 h). Following treatment, media was removed, and cells were washed three times with cold PBS. The next day, each well was digested with 400 µL of 69% HNO_3_ for 1.5 h. Digests were transferred to trace‐metal‐free 15 mL tubes (Labcon, USA), diluted with 7 mL milliQ water, and spiked with 50 ppb ^193^Ir in 2% HNO_3_ as an internal standard. Platinum content was quantified using a Perkin Elmer NexION Inductively Coupled Plasma Mass Spectrometer (ICP‐MS) with an ESI 7‐port valve system (Elemental Scientific, Omaha, USA), following protocols described previously [44]. Instrument performance was optimized daily using supplier‐recommended parameters (Table S2). Results represent the mean ± SEM of three independent experiments run in triplicates and expressed as nmol/10^6^ cells or µM/cell. Platinum uptake was quantified using external standards (Sigma Merck, Sydney, Australia) and a calibration curve (Figure S3).

### 5.6 Mode of Uptake of Fluorouracil‐Platinum(IV) Prodrugs

HT29 cells (1 × 10^6^ cells/well) were seeded into 6‐well plates and incubated overnight to adhere. Cells were then treated with 3 µM of **Pt^IV^P‐5FUMeOBut** or **Pt^IV^56‐5FUMeOBut** under various conditions to investigate cell uptake mechanisms, as previously described [44]. Preliminary experiments informed the selection of incubation times. To assess temperature‐dependent uptake, cells were incubated with the prodrugs for 2 h at either 4°C or 37°C in DMEM. To evaluate transferrin receptor‐mediated uptake, cells were pretreated with 1 µg/mL anti‐transferrin antibody for 2 h, followed by a 2 h incubation with 3 µM of each prodrug at 37°C. for inhibition of clathrin‐mediated endocytosis, cells were pre‐treated with 0.45 M sucrose in serum‐free medium for 30 min before a 2 h exposure to the prodrugs. To block the SLC7A5‐mediated transport, cells were treated with 1 mM *D*‐phenylalanine for 2 h, followed by prodrug incubation for another 2 h. After treatment, the medium was removed, and intracellular platinum levels were measured by ICP‐MS following the protocol in Method 5.5.

### 5.7 Cellular Localization of Fluorouracil‐Platinum(IV) Prodrugs

HT29 cells (1 × 10^6^ cells/well) were seeded into 6‐well plates and incubated overnight to adhere. The next day, cells were treated with 3 µM of either **Pt^IV^P‐5FUMeOBut** or **Pt^IV^56‐5FUMeOBut** for 24 h. Following treatment, media was removed, and cells were washed three times with ice‐cold PBS before being trypsinized. Subcellular fractionation was performed using the Fraction‐PREP Cell Fractionation kit (Abcam, Cambridge, USA) as previously described [44]. Cell pellets were resuspended in ice‐cold PBS and transferred to Eppendorf tubes, followed by centrifugation at 700 × *g* for a 5 min. the pellet was then resuspended in 400 µL of cytosol extraction buffer and incubated on ice for 20 min. After centrifugation (700 × *g* for 10 min), the supernatant was collected as the cytosolic fraction. The remaining pellet was treated with 400 µL of ice‐cold membrane A buffer, vortexed for 10 s, then mixed with membrane B buffer and incubated on ice for 1 min. The sample was vortexed again and centrifuged (1000 × *g*, 5 min), and the resulting supernatant was collected as the membrane/particulate fraction. The remaining pellet was resuspended in 200 µL of cold nuclear extraction buffer, vortexed for 20 s, and incubated on ice for 40 min with intermittent vortexing every 10 min. After centrifugation at maximum speed for 10 mins, the supernatant (nuclear fraction) and pellet (cytoskeletal fraction) were collected separately. All fractions were dried at 100°C, then digested with 180 µL of nitric acid for 1.5 h. Samples were diluted with milli‐Q water to a final volume of 3 mL. In addition, untreated control samples processed through the identical fractionation and ICP‐MS workflow showed platinum levels at or below the LOD, confirming the absence of background contamination from reagents, consumables or the fractionation procedure. Platinum content in each cellular fraction was quantified using ICP‐MS as detailed in Method 5.5. All experiments were performed in triplicate.

### 5.8 Cell Death Analysis

After 72 h of treatment with each agent, apoptosis in HT29 cells was assessed using Annexin V‐FITC and Propidium Iodide (PI) staining (Abcam, Cambridge, USA) following a previously described method [44]. HT29 cells (2 × 10^5^ cells/well) were seeded in 6‐well plates and treated with the respective IC_50_ concentrations (Table 1) for 72 h. After incubation, the supernatant from each well was collected into labelled tubes. Cells were detached using 300 µL of trypsin per well and combined with the corresponding supernatants. Cell viability was assessed via trypan blue exclusion using an inverted microscope (Nikon Eclipse TS100, Sydney, Australia) and the cell suspension was adjusted to 500 cells/µL. Samples were centrifuged at 500× *g* for 5 min at 4°C, and pellets were resuspended in 100 µL of 1× Annexin V Binding Buffer. Each sample was transferred into round‐bottomed polystyrene tubes (Interpath Services, Somerton, Australia), followed by the addition of 2 µL of Annexin V‐FITC and 2 µL of PI. Samples were incubated in the dark for 10 min, then analyzed on a benchtop flow cytometer (Biosciences, Erembodegem, Belgium). Data were visualized using FlowJo v10.9 software as FL1‐H vs. FL2‐H scatter plots. All experiments were conducted in triplicate and repeated three times.

### 5.9 Cell Cycle Arrest

HT29 cells were seeded at 2 × 10^5^ cells/well in 6‐well plates and treated for 72 h with IC_30_ concentration for each investigated agent (cisplatin (IC_30_ 73.95 ± 1.97 µM), 5FU (IC_30_ 61.94 ± 1.87 µM), 5FUMeOBut (IC_30_ 0.42 ± 1.61 µM), **Pt^IV^P‐5FUMeOBut** (IC_30_ 0.30 ± 1.22 µM), **Pt^IV^56‐5FUMeOBut** (IC_30_ 0.15 ± 1.62 µM). After treatment, the culture supernatant was collected and 300 µL of trypsin was added to detach the cells. Detached cells were combined with their respective supernatants, washed twice with PBS, and fixed overnight in 70% ethanol at 4°C. following an additional two PBS washes, cells were stained with a PI solution (50 µg/mL PI in 10 mM Tris‐Cl, pH 8.0, 10 mM NaCl, 0.1% Triton X‐100, and 100 µg/mL RNas) for 45 min. Cell cycle profiles were analyzed using a BD FACSCanto II flow cytometer (Biosciences, Erembodegem, Belgium), and data were processed with FlowJo v10.9 software. All experiments were performed in triplicate across three independent replicates, as previously described [44].

### 5.10 Reactive Oxygen Species (ROS) Detection Assay

The production of ROS in treated cells was assessed using the DCFDA/H_2_DCFDA‐cellular ROS Assay Kit (Abcam, Cambridge, USA), as previously described [24, 44, 124, 155]. HT29 cels (2,500 cells/well) were seeded into 96 well plates and incubated overnight. After washing with 1× assay buffer, cells were incubated with 25 µM 2′,7′‐dichlorofluorescein diacetate (DCFH‐DA) for 45 min. The dye was then removed, and cells were washed again with 1× assay buffer before being overlaid with phenol red‐free medium. Treatments were applied at their respective IC_50_ concentration (Table 1) for 72 h for each prodrug, CLB, or cisplatin. Fluorescence intensity for relative fluorescence units (RFU) was measured at 485/535 nm using a Glo‐Max‐Multimode plate reader (Promega Corporation, Alexandra, Australia) at various time points. For the positive control, cells were stained with DCFDA, followed by treatment with 20 µM tert‐butyl hydroperoxide (TBHP) before fluorescence measurement. Three independent experiments were conducted, with each sample run in triplicates.

### 5.11 Mitochondrial Membrane Potential

Changes in MtMP were assessed using the TMRE‐MtMP Assay Kit (Abcam, Cambridge, USA), as previously described [38, 44, 155]. HT29 cells (2,500 cells/well) were seeded into 96‐well plates and treated with the IC_50_ concentration for each prodrug, 5FU, 5FUMeOBut or cisplatin (Table 1). At 24, 48, or 72 h post‐treatment, cells were washed with PBS and stained with 1 µM tetramethylrhodamine, ethyl ester (TMRE) for 30 min. After staining, cells were washed three times with PBS containing 0.2% BSA, followed by the addition of phenol‐red free DMEM. Fluorescence was measured using Glo‐Max Multimode plate reader (Promega Corporation, Alexandra, Australia) at 549/575 nm with results expressed as RFU. As a positive control, 20 µM carbonyl cyanide 4‐(trifluoromethoxy) phenylhydrazone (FCCP) was added to the cells, incubated for 10 min, and then stained with TMRE as described above. Three independent experiments were conducted, with each sample was run in triplicates.

### 5.12 Immunofluorescence Morphological Changes in Microtubule Organization Using Confocal Microscopy

Millicell 8‐well chamber slides (Merck, Darmstadt, Germany) were seeded with 1,000 HT29 cells/well to examine the changes in actin and tubulin expression and cell morphology following treatment, as previously described [44]. Cells were treated with IC_50_ concentration (Table 1) of each investigated agent for 72 h. After treatment, cells were washed with PB (75 mM disodium phosphate, 25 mM monosodium phosphate, pH. 7.4) for 5 min. Samples were fixed with 200 µL of 4% paraformaldehyde for 10 min, followed by permeabilization with 200 µL of 0.2% Triton X‐100 in PBS for 10 min. Nonspecific binding was blocked with 1% BSA (Sigma–Aldrich) in PB for 30 min. Cells were then washed with PBS and twice with 0.1% PBS‐Tween before incubation with primary conjugated antibodies for 1 h at room temperature: β‐tubulin (Rabbit mAb (9F3), Alexa Fluor(R) 488 Conjugate, Cell signaling, Danvers, USA) and phalloidin (iFluor 555 reagent, ab176756; Abcam, Cambridge, USA). Following incubation, cells were washed twice with 0.1% PBS‐Tween. The chambers were removed, and slides were mounted with ProLong Gold Antifade Mountant with DAPI (ThermoFisher, Eugene, USA). Coverslips were sealed and air‐dried. Imaging was performed using a Zeiss LSM 800 confocal microscope. Fluorescence intensity measurements were acquired at 20× magnification with consistent imaging parameters across all conditions analyzed using CellProfiler software 4.2.5. For morphological analyses, images were acquired at 63× magnification using the Airyscan detector and processed with the Zen Blue Airyscan processing module. A total of 30 cells (*n* = 30) were analyzed for each condition.

### 5.13 Wound Healing Assay

HT29 cells were seeded at a density of 10^5^ cells/well in an Incucyte Imagelock 96‐well plate (Sartorius, Gottingen, Germany) and incubated overnight. Wounds were simultaneously generated using the Incucyte 96‐Well ESSEN Bioscience woundmaker tool (Sartorius, Gottingen, Germany) as previously described [44, 156, 157, 158, 159, 160]. Following wound formation, the medium was removed, and cells were washed with PBS. Fresh DMEM was added, and cells were treated with the IC_50_ concentration (Table 1) of each investigated agent for 72 h. The plate was then incubated in the Incucyte Live‐Cell analysis system for real‐time imaging over 72 h (objective: 10×; channel: phase contrast; scan type: scratch wound; scan interval: every 4 h). Data were processed and analyzed using the Incucyte Scratch Wound Analysis Software Module (Cat. No. 9600–0012). Three independent experiments were conducted, and each condition run in triplicate.

### 5.14 Western Blot Analysis

HT29 cells were seeded in 6‐well plates at 2 × 10^5^ cells/well and treated with the IC_50_ concentration (Table 1) of each investigated agents for 72 h. Supernatants were collected and centrifuged at 500× *g* for 5 min at 4°C. Meanwhile, total protein was extracted from the adherent cells using 200 µL of RIPA lysis buffer, and protein concentrations were quantified using the Bio‐Rad protein assay (Bio‐Rad, Hercules, USA) as previously described [44]. Equal amounts of protein were separated by sodium dodecyl‐sulfate polyacrylamide gel electrophoresis (SDS‐PAGE) using Bolt 4%–12% Bis‐Tris gel (ThermoFisher, Eugene, USA), run at 80 V for 30 min followed by 120 V for 60 min with PageRuler Prestained Protein Ladder or SeeBlue Prestained Protein Standard (Invitrogen #LC5625). Proteins were transferred to 0.2 µm PVDF membrane Cytiva, Amersham, UK) using semi‐dry blot transfer for 30 min. Membranes were blocked with 5% BSA in TBST for 1 h and then incubated overnight at 4°C with primary antibodies targeting apoptotic, anti‐apoptotic, proliferative, and autophagic markers, according to manufacturer's protocol, with antibody details and molecular weights listed in Table S6. After three 10 min TBST washes, membranes were incubated with either mouse or rabbit HRP‐conjugated secondary antibodies (1/5000) for 90 min. Protein bands were visualized using enhances chemiluminescence (ECL) kit (ThermoFisher, Eugene, USA), and images were acquired with the Odyssey FC imaging system (LI‐COR Biosciences, Lincoln, USA). Band intensities were normalized to GAPDH and to their corresponding no‐treatment control using ImageJ v1.53t (National Institutes of Health, Bethesda, USA).

### 5.15 Proteomics

HT29 cells were seeded at 2 × 10^5^ HT29 cells/well in 6‐well plates and treated with IC_50_ concentration (Table 1) of the platinum complexes for 72 h for proteomic analysis as previously described [44]. The supernatant was collected, and adherent cells were detached with trypsin. Cells were centrifuged at 500 × *g* for 5 min at 4°C, washed with PBS, and resuspended in 300 µL RIPA buffer on ice for 30 min. Lysates were centrifuged at 12,000× *g* for 15 min at 4°C, and the supernatants were collected in Lo‐Bind Eppendorf tubes. Protein concentrations were determined using the Bio‐Rad protein assay.

Protein extraction and de‐lipidation were performed by adding 450 µL methanol to 100 µL of cell lysate, followed by vortexing. Next, 150 µL chloroform was added and vortexed, then 450 µL water was added, followed by vortexing and centrifugation at 12,000× *g* for 5 min to form a protein pellet at the organic/aqueous interface. The chloroform layer was removed, and 400 µL methanol was added, vortexed, and centrifuged again at 12,000× *g* for 15 min. the supernatant was discarded, and the pellet was washed with 400 µL methanol, then air‐dried overnight.

Proteins were solubilized with 20 µL of 0.1% RapiGest in 50 mM ammonium bicarbonate and vortexed. Reduction was performed with 5 mM DTT in 50 mM ammonium bicarbonate at 60°C for 30 min, followed by alkylation with 15 mM iodoacetamide at room temperature in the dark for 30 mins. Proteins were digested overnight at room temperature with Trypsin Gold (10 ng/µL; Promega, Sydney, Australia). The next day, 50 µL of 4% trifluoroacetic acid was added to each sample and incubated at 37°C for 45 min to degrade RapiGest. Samples were centrifuged at 12,000× *g* for 10 min at 4°C, and the supernatants were transferred to total recovery vials.

Samples were analyzed using a nanoAcquity UPLC system coupled to a Synapt G2‐Si mass spectrometer (Waters, Milford, USA) for label‐free quantitative proteomic profiling. Full details of the nano‐proteomics method are provided in Method S2. Three independent experiments were conducted in triplicate, and all samples were analyzed in a single run with a single 1 µL injection per trial. Mass spectrometry data were processed using Progenesis QI for proteomics (version 4.1, Nonlinear Dynamics, Newcastle upon Tyne, UK), as previously described [44].

### 5.16 Statistical Analysis

Data were presented as Mean ± SEM from three independent experiments. Statistical tests used for each dataset are specified in the corresponding Figure legends. Differences between groups were considered statistically significant at *p* < 0.05. Significance is indicated as follows: *p < 0.05, **p < 0.01, ***p < 0.001, and ****p < 0.0001, unless otherwise stated.

## 6 Patents

This work is part of Australian Provisional Patent Application 2022900110, Platinum(IV) complexes, February 2022, Western Sydney University, Sydney, Australia.

## Author Contributions

Conceptualization, M.G.E., K.F.S., and J.R.A‐W.; methodology, M.G.E., S.F., T.J.M., S.K., M.M., K.F.S. and J.R.A‐W.; complex synthesis, M.G.E. and A.K.; software, M.G.E., S.F., T.J.M., S.K. and M.M.; validation, M.G.E.; formal analysis, M.G.E.; investigation, M.G.E.; data curation, M.G.E.; writing – original draft preparation, M.G.E.; writing – review and editing, M.G.E., P.d.S., C.P.G., K.F.S., and J.R.A.‐W.; visualization, M.G.E.; supervision, K.F.S., and J.R.A.‐W. All authors have read and agreed to the published version of the manuscript.

## Funding

The authors thank Western Sydney University for its financial support. M.G.E. was supported through an Australian Postgraduate Award.

## Institutional Review Board Statement

Not applicable.

## Informed Consent Statement

Not applicable.

## Conflicts of Interest

The authors declare no conflicts of interest.

## Supporting information



The following Supporting Information can be downloaded at: www.
**Supporting File 1**: chem70835‐sup‐0001‐SuppMat.docx.

## Data Availability

All data relevant to the publication are included.
