# Supplementary material for: Chemotherapeutic Potential of Fluorouracil‐Platinum (IV) Prodrugs Against Cisplatin‐Resistant Colorectal Cancer Cells
Source: Chemistry. 2026 Feb 27;32(30):e03422. doi: 10.1002/chem.202503422 (PMC13472338; doi:10.1002/chem.202503422)
Supplement: Supplementary file 1 [file CHEM-32-e03422-s001.docx]

**Supplementary**

Chemotherapeutic Potential of Fluorouracil-Platinum(IV) Prodrugs Against Cisplatin-Resistant Colorectal Cancer Cells

Maria George Elias^1,2,3^, Aleen Khoury^1^, Shadma Fatima^3^, Timothy J. Mann^2,3^, Shawan Karan^1^,
Meena Mikhael^4^, Paul de Souza^5^, Christopher P. Gordon^1^, Kieran F. Scott^2,3^ and Janice R. Aldrich-Wright^1,2,^*

Faculty of Engineering, Computing and Science, Western Sydney University, Sydney, NSW 2751, Australia; m.elias3@westernsydney.edu.au (M.G.E.); aleen.khoury@westernsydney.edu.au (A.K.); s.fatima@westernsydney.edu.au (S.F.); shawan.karan@westernsydney.edu.au (S.K.); c.gordon@westernsydney.edu.au (C.P.G.)

^2^	Faculty of Health, School of Medicine, Western Sydney University, Sydney, NSW 2751, Australia; kieran.scott@westernsydney.edu.au (K.F.S.)

^3^	Medical Oncology, Ingham Institute for Applied Medical Research, Liverpool Hospital,
Liverpool, NSW 2170, Australia; tim.mann@unsw.edu.au (T.J.M.)

^4^	Mass Spectrometry Facility, Western Sydney University, Sydney, NSW 2751, Australia;
m.mikhael@westernsydney.edu.au (M.M.)

^5^	Nepean Clinical School, Faculty of Medicine and Health, University of Sydney,
Kingswood, NSW 2747, Australia; paul.desouza@sydney.edu.au

*****	Correspondence: j.aldrich-wright@westernsydney.edu.au; Tel.: +61-2-4620-3218

**Method S1. Syntheses of Fluorouracil-platinum(IV) prodrugs**

**The general syntheses of 5-fluorouracil-1-methoxy-4-butanoic acid**

The 5-fluorouracil-1-methoxy-4-butanoic acid (5FUMeOBut) was synthesised as previously reported.^1^ Using formaldehyde solution (37%) (0.52 mL, 6.9 mmol), 5-fluorouracil (5FU) (500 mg; 3.80 mmol) was dissolved. The solution was stirred for 5 h at 60 ^o^C. The solution was then reduced under vacuum into a thick transparent liquid. To this liquid, 4 mL of ACN, succinic anhydride (577 mg, 5.80 mmol) and DMAP (catalytic amount) was added and stirred at 50 ^o^C for 36 h. The reaction mix was then reduced under vacuum and purified by means of flash chromatography (Biotage Isolera^TM^) set with a Biotage^®^ SNAP C18-reverse phase column (KP- C18-HS 12g). The mobile phase consisted of solvents, A (d.i.H_2_O) and B (MeOH). Each sample was dissolved with d.i.H_2_O/MeOH (50:50) and eluted through the column with H_2_O:MeOH gradient (0% B for 10 column volumes and 0 – 14% for 8 column volumes) with a flow rate of 3 mL/min, collected within the set wavelength range of 200 – 400 nm.

**The general syntheses of [Pt(P_L_)(1S,2S-diaminocyclohexane)Fluorouracil(OH)](NO_3_)_2_**

[Pt(P_L_)(1*S*,2*S*-diaminocyclohexane)(OH)_2_]^2+^ ^2^ was coordinated to 5FUMeOBut, a bioactive ligand (Bx), by substitution of either one of the hydroxido axial ligands to obtain [Pt(P_L_)(1*S*,2*S*-diaminocyclohexane)Bx(OH)]^2+^.

[Pt(P_L_)(1*S*,2*S*-diaminocyclohexane)(OH)_2_]^2+^ was reacted with 5FUMeOBut (0.50 mmol) in DMSO (1-2 mL), NHS (1.2 mol eq.) and DCC (1.2 mol eq.), and the mix was stirred at RT overnight. This resulted in a by-product dicyclohexylurea (DCU), which was insoluble. The DCU was filtered out. The filtrate was then added to [Pt(P_L_)(1*S*,2*S*-diaminocyclohexane)(OH)_2_]^2+^ (0.30 mmol) and stirred at RT for 2 h in the dark. The solution was then diluted with 4 mL of MeOH and then precipitated with 40 mL of ether. A white precipitate formed [Pt(P_L_)(1*S*,2*S*-diaminocyclohexane)(5FUMeOBut)(OH)]^2+^. Using vacuum filtration, the precipitate was collected and washed with ether twice. Purification of the 5FU-platinum(IV) prodrugs (**Pt^IV^P- 5FUMeOBut** or **Pt^IV^56-5FUMeOBut**; Figure 1) was carried out using the flash chromatography system.^2^ Each sample was eluted through the column with varying H2O:MeOH gradients (0% B for 10 column volumes and 0 – 15% B for 6 column volumes) with a flow rate of 3 mL/min, collected within the set wavelength range of 200 – 400 nm. The successful synthesis and purity of the 5FU-platinum(IV) prodrugs was confirmed by high performance liquid chromatography (HPLC), ^1^H nuclear magnetic resonance (^1^H NMR), ^1^H-^195^Pt heteronuclear multiple quantum coherence (^1^H-^195^Pt-HMQC) NMR and circular dichroism (CD) spectroscopy.^2^

HPLC chromatograms were determined by eluting the 5FU-platinum(IV) prodrugs through an Agilent Technologies Infinity HPLC machine, equipped with a Phenomenx Onyx^TM^ Monolithic C_18_-reverse phase column (100 × 4.6 mm, 5 μm pore size). For organic compounds an Agilent ZORBAX RX-C_18_ column (100 × 4.6 mm, 3.5 μm pore size) was used. The mobile phase consisted of solvents, A (0.06% trifluoroacetic acid (TFA) in deionised water (d.i.H_2_O)) and B (0.06% TFA in ACN/d.i.H_2_O (90:10)). An injection volume of 5 μL was utilised and eluted with a 0 – 100% linear gradient over 15 min with a flow rate of 1 mL/min, within the set wavelengths of 214 and 254 nm. ^1^H NMR was determined for each prodrug by dissolving the prodrug (5 mg) in deuterium oxide (D_2_O) (500 µL). ^1^H NMR was carried out on a 400 MHz Bruker Avance Spectrometer at 298 K. All samples were prepared to a concentration of 10 – 20 mM in 450 – 600 μL using D_2_O. ^1^H NMR was set to 10 ppm and 16 scans with a spectral width of 8250 Hz and 65536 data points. ^1^H-^195^Pt HMQC was carried out using a spectral width of 214436 Hz and 256 data points for ^195^Pt nucleus, F1 dimension, also a spectral width of 4808 Hz with 2048 data points for ^1^H nucleus, F2 dimension. All resonance recorded were presented as chemical shifts in parts per million (δ ppm). A Jasco J-810 CD spectrophotometer was used to measure the CD spectra of the purified platinum(IV) prodrugs. The samples were prepared in d.i.H_2_O, with a concentration of 0.05 mM and a 1 mm optical glass cuvette was used. CD experiments were performed in the range of 350–200 nm (20 accumulations) with a bandwidth of 1 nm, data pitch of 0.5 nm, a response time of 1 sec, and a 100 nm/min scan speed. The flowrate of nitrogen gas was 8 L/min. Excel was used to process the spectral data.

**Result S1.** **Characterisation and yields of 5-fluorouracil-1-methoxy-4-butanoic acid** **and Fluorouracil-platinum(IV) prodrugs**

HPLC, NMR (^1^H and ^1^H-^195^Pt-HMQC) and CD were utilised to confirm the structures and purity of the 5FU-platinum(IV) prodrugs for biological testing, passing for greater than 95% purity (Table H.S1). The obtained yields of the 5FU-platinum(IV) prodrugs are reported in Table H.S1 and HPLC chromatograms, which include the retention time (t_R_) of the prodrugs are also reported in Figures H.S2-S3. The obtained yield of the 5FUMeOBut was 520 mg, HPLC t_R_: 6.017 min; purity 97.7% (Figure H.S1). The obtained ^1^H and ^1^H-^195^Pt-HMQC NMR are tabulated in Table N.S1 and the spectra are reported in Figures N.S1–S5. The HPLC chromatograms (Figure H.S1-3) and NMR spectra (Figure N.S1-8) matched those of literature data, which confirmed the successful syntheses and isolation of the products of interest.^1^ The CD experiments confirmed the retention of chirality of the 1*S*,2*S*-diaminocyclohexane. The obtained results for the 5FU-platinum(IV) prodrugs were comparable to previously reported data.^1^ All the characteristic peaks from CD spectra of the purified complexes are summarised in Table H.S1.

**Table H.S1.** Summary of the characterisation data of 5FUMeOBut ligand and the 5FU-platinum(IV) prodrugs.

| **Complexes** | **Yields [mg]** | **Yields [%]** | **t_R_ [min]** | **Purity [%]** | **CD λ_max_ [nm] (mdeg.mol / L × 10^1^)** |
| --- | --- | --- | --- | --- | --- |
| **5FUMeOBut** | 520 | 63 | 6.017 | 97.7 | – |
| **Pt^IV^P-5FUMeOBut** | 227 | 83 | 7.888 | 98.4 | 208 (–340.04), 252 (–17.84), 273 (–67.74), 290 (–35.73) |
| **Pt^IV^56-5FUMeOBut** | 193 | 75 | 9.258 | 96.6 | 208 (–300.75), 255 (+9.87), 290 (–42.4) |

**Table N.S1.** Summary of NMR spectroscopy data of 5FU-platinum(IV) prodrugs, showing the chemical shifts (ppm), integration, multiplicity and coupling constants.^[a]^

| **Proton Labels** | **Complexes** | | |
| --- | --- | --- | --- |
|  | **5FUMeOBut** | **Pt^IV^P-5FUMeOBut** | **Pt^IV^56-5FUMeOBut** |
| H2/H9^[b]^ | – | 9.27 (d, 1H, *J* = 5.5 Hz) | 9.21 (m, 4H) |
| H4/H7 | – | 9.09 (d, 2H, *J* = 8.32 Hz) |  |
| H5/H6 | – | 8.33 (s, 2H) | – |
| H3/H8 | – | 8.26 (t, 2H, *J* = 7.03 Hz, 2H) | 8.24 (t, 2H, *J* = 7.10 Hz) |
| A/B | 2.61 (s, 4H) | 2.24 (m, 4H) | 2.21 (m, 4H) |
| C | 5.88 (s, 2H) | 5.27 (s, 2H) | 5.31 (s, 2H) |
| D | 7.61 (d, *J* = 5.11 Hz, 1H) | 7.61 (d, 1H, *J* = 5.08 Hz) | 7.59 (d, 1H, *J* = 5.09 Hz) |
| CH_3_ | – | – | 2.83 (s, 6H) |
| H1'/H2' | – | 3.18 (m, 2H) | 3.17 (m, 2H) |
| H3’/H6’ equatorial | – | 2.40 (m, 2H) | 2.39 (m, 2H) |
| H4’/H5’ equatorial and H3’/H6’ axial | – | 1.69 (m, 4H) | 1.68 (m, 4H) |
| H4’/H5’ axial | – | 1.29 (m, 2H) | 1.30 (m, 2H) |
| ^1^H/^195^Pt | – | 9.29/535  8.26/535 | 9.20/545  8.24/545 |

[a] Experiments were performed in DMSO-d_6_ or D_2_O. Accordingly, ammine or hydroxido resonances are not observed due to proton exchange. [b] Symmetrical complexes, thus H2/9, H3/8, H4/7 and H5/6 are equivalent protons.

**HPLC Chromatograms**

**Figure H.S1.** The HPLC chromatogram of 5FUMeOBut within the collection wavelength of 254 nm acquired at 298 K by an Agilent ZORBAX RX-C18 column (100 × 4.6 mm, 3.5 μm pore size). t_R_ at 6.017 min.

**Figure H.S2**. The HPLC chromatogram of **P-5FUMeOBut**, at 254 nm and 298K. Phenomenex Onyx^TM^ Monolithic C18-reverse phase column (100 × 4.6 mm, 5 μm pore size) was used at a gradient of 0–100% (ACN:H_2_O, 9:1) over 15 min. t_R_ at 7.888 min.

**Figure H.S3.** The HPLC chromatogram of **56-5FUMeOBut**, at 254 nm and 298K. Phenomenex Onyx^TM^ Monolithic C18-reverse phase column (100 × 4.6 mm, 5 μm pore size) was used at a gradient of 0–100% (ACN:H_2_O, 9:1) over 15 min. t_R_ at 9.258 min.

**^1^H NMR and ^1^H-^195^Pt HMQC Spectra**

**Figure N.S1.** ^1^H NMR spectra of 5FUMeOBut in deuterated DMSO obtained at 298 K. Inset: structure of 5FUMeOBut, with proton labelling system.

**Figure N.S2.** ^1^H NMR spectra of **Pt^IV^P-5FUMeOBut** in D_2_O obtained at 298 K. Inset: structure of **Pt^IV^P-5FUMeOBut**, with proton labelling system.

**Figure N.S3.** ^1^H-^195^Pt HMQC spectra of **Pt^IV^P-5FUMeOBut** in D_2_O obtained at 298 K.

**Figure N.S4.** ^1^H NMR spectra of **Pt^IV^56-5FUMeOBut** in D_2_O obtained at 298 K.

**Figure N.S5.** ^1^H-^195^Pt HMQC spectra of **Pt^IV^56-5FUMeOBut** in D_2_O obtained at 298 K.

**^13^C NMR Spectra**

**Figure N.S6.** ^13^C NMR spectrum of 5FUMeOBut in D_2_O. Insert: The chemical structure of 5FUMeOBut, with carbon labelling system.

**Figure N.S7.** ^13^C NMR spectrum of **Pt^IV^P-5FUMeOBut** in D_2_O. Insert: The chemical structure of **Pt^IV^P-5FUMeOBut**, with carbon labelling system.

**Figure N.S8.** ^13^C NMR spectrum of **Pt^IV^56-5FUMeOBut** in D_2_O. Insert: The chemical structure of **Pt^IV^56-5FUMeOBut**, with carbon labelling system.

**Method S2. Nano Proteomics Method**

The digested protein samples were analysed by UPLC-MS using a Waters nanoAcquity UPLC sample manager fitted with a binary solvent manager. Mass spectrometric detection was conducted using a Waters Synapt G2-Si. Separation consisted of two mobile phases. Mobile Phase A (0.1% formic acid in Milli-Q water) and Mobile Phase B (0.1% formic acid in ACN). The trapping column was a Waters nanoEase M/Z Symmetry C18 trap column (180 µm x 20 mm) and the analytical column was a Waters nanoAcquity UPLC 1.7 μm BEH130 C18 column (75 µm x 100 mm) thermostatted to 40 °C. Elution was achieved at a flow rate of 0.3 µL/min with each sample run for 55 minutes. The gradient was 0 min 1% B; 2 min 10% B; 40 min 40% B; 42 min 85% B and 50 min 85% B. System specific cleaning and equilibration protocols were run before each sample.

Mass spectrometry was conducted in positive ion mode with a capillary voltage of 3 kV and a sampling cone voltage of 30 V as well as a source offset of 30 V for electrospray ionizations. The source temperature was set at 80 °C. A desolvation source of nitrogen gas at 20 L/h and a desolvation temperature of 350 °C was used. Lock spray ion acquisition was conducted every 300 seconds with [Glu1]-fibrinopeptide B as the reference compound. Data acquisition was conducted over the mass to charge range of 50–2000. The data independent acquisition used an HDMSe experiment employing both low and high energy collision-induced dissociation of parent ions. Low energy collision was done at 6 V in the trap collision cell and at 4 V in the transfer collision cell. High energy collision used a collision energy ramp from 17 to 60 V in the transfer collision cell. Scan time was 0.5 seconds and after each scan the system would switch from high to low energy collision.

Protein identification was carried out using Progenesis QI for Proteomics and the UniProt Homo Sapiens reference database with the following conditions. The parent and fragment ion tolerances were set to automatic, the allowed maximum missed cleavages was set to 1, the allowed false discovery rate was set to 4% and the maximum protein size was set to 250 kDa.  The peptide modifications were carbamidomethyl C (fixed) and oxidation M (variable). The ion matching requirements were fragments/peptide of 1 or more, fragments/protein of 3 or more and peptides/protein of 1 or more.  Relative protein quantitation was performed with the Hi-N method using the top 3 peptides. Progenesis QI automatically selects unique reporter peptides to quantify a protein and measures their UPLC peak areas in the total ion chromatogram.

**Table S1.** **Matrix Conditions selected for RASTRUM bioprinting.**

| **Matrix Code** | **Formulation** | **Stiffness (kPa, storage modulus)** | **F-code (Bioink)** | **F-code (Activator)** |
| --- | --- | --- | --- | --- |
| Px02.09 | GFOGER, RGD | 1.1 | F236 | F177 |

**Figure S1. Effect of fluorouracil-platinum(IV) prodrugs on the survival of cancer, normal epithelial cells and spheroids.** A. MCF10A, B. HT29 Cells, and C. HT29 spheroids were treated with 3-fold dilutions of the different agents including cisplatin, 5FU, 5FUMeOBut, **Pt^IV^P-5FUMeOBut** or **Pt^IV^56-5FUMeOBut** starting with a concentration of 150 μM and assayed for cell viability as described in Method 4.3 and 4.4. The data for cisplatin was obtained from previously reported data run in parallel with these experiments.^2^ Data points denote mean ± SEM from three independent experiments where samples were run in triplicates.

**Figure S2. HT29 spheroids.** Cell viability following 72 h treatment with **Pt^IV^P-5FUMeOBut** and **Pt^IV^56-5FUMeOBut** prodrugs, as well as 5FU, 5FUMeOBut, and cisplatin in HT29 spheroids. Bright-field live images were acquired using an Incucyte® phase-contrast microscope with a 10× objective. HT29 spheroids were generated using a non-contact drop-on-demand 3D RASTRUM bioprinter (Inventia Life Science), as described in Methods Section 5.4, producing uniform spheroids in a polyethylene glycol-based bioink that were cultured for one week prior to drug treatment. Representative micrographs show spheroids after 72 h exposure to the indicated compound concentrations (150, 0.6713, and 0.0006 μM).

**Figure S3. Calibration curve generated by plotting the peak areas measured by the ICP-MS against known concentrations (^195^Pt(STD)).** This curve was used to quantify the cellular uptake of the Pt complexes; y=0.007x + 0.000 and R^2^= 0.999997

**Table S2. Conditions and parameters selected on the ICP-MS machine**.

| **Parameter** | **Value** |
| --- | --- |
| Plasma RF power | 1500 W |
| Nebulizer gas flow rate | 0.92-0.94 L.min^-1^ |
| Auxiliary gas flow rate | 1.2 L.min^-1^ |
| Collision gas flow rate (He) | 4.5 L.min^-1^ |
| KED Cell Entrance/Exit voltage | -8/-25 V |
| KED CRO/QRO Voltage | -15/-12 V |
| Deflector Voltage | -9V |
| Isotope monitored | ^195^Pt |
| Dwell times | 50 ms |
| Integration Time | 4500 ms |

RF, radio frequency; He, helium; KED, kinetic energy discrimination

**Figure S4.** ICP-MS analysis for the uptake of **Pt^IV^P-5FUMeOBut** and **Pt^IV^56-5FUMeOBut** in HT29 at 0, 0.5, 1, 3, 6, 12, 24 and 30 h. The **Pt^IV^56-5FUMeOBut** prodrug has the greatest cellular uptake in HT29. *n* = 3 from three independent experiments where samples were run in triplicates. Data points denote mean ± SEM and expressed in $\mu$M/ cell.

**Table S3. Kinetic Cellular uptake of** **Pt^IV^P-5FUMeOBut** **and** **Pt^IV^56-5FUMeOBut** **prodrugs in HT29.** Table shows the average value of cellular concentration in nmol/10^6^ cells (top) and the ratio of intracellular concentration to extracellular concentrations (bottom) for each complex. *n* = 3 from three independent experiments where samples were run in triplicates. Data points denote mean ± SEM.

|  |  | **Time (h)** | | | | | | | |
| --- | --- | --- | --- | --- | --- | --- | --- | --- | --- |
|  | **Complex** | **0** | **0.5** | **1** | **3** | **6** | **12** | **24** | **30** |
| **Intracellular Concentration  (nmol/10^6^ cells)** | **Pt^IV^P-5FUMeOBut** | 0.12  ± 0.05 | 0.21  ± 0.12 | 0.29  ± 0.05 | 0.95  ± 0.49 | 0.85  ± 0.28 | 9.43  ± 1.13 | 10.49  ± 2.34 | 12.87  ± 3.01 |
|  | **Pt^IV^56-5FUMeOBut** | 0.25  ± 0.08 | 0.45  ± 0.12 | 0.71 ±0.13 | 1.52 ±0.58 | 2.59 ±1.15 | 14.92 ±3.73 | 22.21 ±5.95 | 23.51  ± 5.61 |
|  | **Cisplatin** | 0.36 ± 0.04 | 0.94 ± 0.55 | 0.14 ± 0.02 | 0.28 ± 0.09 | 0.10 ± 0.01 | 0.57 ± 0.23 | 1.02 ± 0.57 | 0.71 ± 0.18 |
| **Intracellular/ extracellular**  **ratio** | **Pt^IV^P-5FUMeOBut** | 18.09 ±15.23 | 33.68  ±55.98 | 46.18  ± 24.48 | 148.64  ± 232.82 | 133.53  ±228.02 | 1480.97  ±530.67 | 1648.09  ±1101.45 | 2021.91  ±1420.77 |
|  | **Pt^IV^56-5FUMeOBut** | 39.20  ± 37.61 | 70.43  ±54.29 | 111.82 ±103.87 | 239.37 ±277.47 | 408.44 ±541.94 | 2345.59 ±1758.03 | 3489.22 ±2805.36 | 3693.61  ±2640.03 |
|  | **Cisplatin** | 57.31 ± 20.91 | 147.74 ±258.72 | 22.65 ± 9.82 | 43.31 ± 46.90 | 16.07 ± 6.78 | 89.42 ± 106.10 | 160.54 ± 271.72 | 112.14 ± 83.99 |

**Figure S5. Mode of uptake of Pt^IV^P-5FUMeOBut** **and** **Pt^IV^56-5FUMeOBut in HT29.** The intracellular amount of Pt was measured by ICP-MS after incubation at 37 °C or 4 °C, as well as following inhibition of clathrin-mediated endocytosis, SLC7A5 receptor or transferrin receptor. Data denote mean ± SEM of three independent experiments where samples were run in triplicates and expressed in $\mu$M/cell. **p* < 0.05, ***p* < 0.01, ****p* < 0.001 and *****p* < 0.0001 in comparison to the optimal conditions.

**Figure S6. Cellular Localization of Pt^IV^P-5FUMeOBut** **and** **Pt^IV^56-5FUMeOBut in HT29 cells.** Platinum concentration in each isolated subcellular fraction was measured by ICP-MS following cellular fractionation and normalised to cell number (reported as µM/cell). Because each fraction is extracted and resuspended in a distinct volume, the reported values reflect fraction-specific platinum enrichment and are not directly additive across fractions. cellular fractionation. Data denote mean ± SEM of three independent experiments where samples were run in triplicates. **p* < 0.05, ***p* < 0.01, ****p* < 0.001 and *****p* < 0.0001 compared with the indicated fractions.

**Figure. S7.** ROS production at 0, 0.25, 0.5, 1, 3, 6, 12, 24, 48 and 72 hours in HT29 upon treatment with 5FU, 5FUMeOBut, **Pt^IV^P-5FUMeOBut**, **Pt^IV^56-5FUMeOBut,** cisplatin and TBHP: t-butyl hydroperoxide. Data points denote mean ± SEM. *n* = 3 from three independent experiments where samples were run in triplicate.

**Table S4.** ROS production upon treatment with 5FU, 5FUMeOBut, **Pt^IV^P-5FUMeOBut**, **Pt^IV^56-5FUMeOBut**, **Pt^II^PHEN*SS***, **Pt^II^56ME*SS***, cisplatin and TBHP: t-butyl hydroperoxide in HT29 cells at 24, 48 and 72 h.

| Complex | **ROS production in different time intervals (RFU)** | | |
| --- | --- | --- | --- |
|  | **HT29** | | |
|  | **24 h** | **48 h** | **72 h** |
| Control | 57.22 ± 0.34 | 63.22 ± 0.30 | 67.33 ± 0.21 |
| Cisplatin | 205.39 ± 11.26 | 285.11 ± 6.49 | 316.55 ± 5.21 |
| TBHP | 544.67 ± 9.04 | 369.22 ± 2.14 | 371.44 ± 1.74 |
| **Pt^II^PHEN*SS*** | 219.00 ± 2.52 | 256.22 ± 3.67 | 273.00 ± 3.71 |
| **Pt^II^56ME*SS*** | 321.56 ± 4.44 | 433.67 ± 3.65 | 453.89 ± 4.91 |
| 5FU | 163.44 ± 2.12 | 199.00 ± 3.85 | 211.56 ± 4.06 |
| 5FUMeOBut | 166.55 ± 4.77 | 238.78 ± 3.04 | 281.44 ± 2.71 |
| **Pt^IV^P-5FUMeOBut** | 247.55 ± 2.13 | 319.78 ± 2.06 | 379.11 ± 3.25 |
| **Pt^IV^56-5FUMeOBut** | 315.56 ± 5.57 | 378.33 ± 5.25 | 503.00 ± 4.18 |

The data for cisplatin, **Pt^II^PHEN*SS*** and **Pt^II^56ME*SS*** was obtained from previously published data run in parallel. ^44^

**Table S5.** Mitochondrial membrane potential upon treatment with 5FU, 5FUMeOBut, **Pt^IV^P-5FUMeOBut**, **Pt^IV^56-5FUMeOBut**, **Pt^II^PHEN*SS***, **Pt^II^56ME*SS***, cisplatin and FCCP in HT29 cells at 24, 48 and 72 h.

| Complex | **MtMP at different time intervals (RFU)** | | |
| --- | --- | --- | --- |
|  | **HT29** | | |
|  | **24 h** | **48 h** | **72 h** |
| Control | 471.52 ± 19.62 | 398.14 ± 15.77 | 409.06 ± 6.18 |
| Cisplatin | 270.02 ± 10.56 | 237.68 ± 4.61 | 188.59 ± 17.95 |
| FCCP | 184.32 ± 9.51 | 143.31 ± 4.61 | 83.58 ± 3.99 |
| **Pt^II^PHEN*SS*** | 321.85 ± 21.92 | 256.63 ± 8.58 | 236.73 ± 10.29 |
| **Pt^II^56ME*SS*** | 159.93 ± 9.67 | 151.44 ± 13.23 | 102.76 ± 7.13 |
| 5FU | 261.14 ± 9.88 | 247.85 ± 33.07 | 202.45 ± 13.25 |
| 5FUMeOBut | 268.70 ± 17.92 | 253.38 ± 7.11 | 216.98 ± 21.26 |
| **Pt^IV^P-5FUMeOBut** | 217.38 ± 29.44 | 211.75 ± 10.96 | 146.35 ± 11.29 |
| **Pt^IV^56-5FUMeOBut** | 209.45 ± 27.81 | 161.51 ± 2.35 | 124.85 ± 16.74 |

The data for cisplatin, **Pt^II^PHEN*SS*** and **Pt^II^56ME*SS*** was obtained from previously published data run in parallel. ^44^

**Figure S8.** Effect of 5-fluorouracil-platinum(IV) prodrugs on β-tubulin and F-actin. Immunofluorescence upon treatment with **Pt^IV^P-5FUMeOBut** and **Pt^IV^56-5FUMeOBut** prodrugs, as well as 5FU, 5FUMeOBut ligands and cisplatin in HT29. Airyscan images were collected at 63×. Confocal microscope paramters were constant across all treatments for comparison in expression. *n* = 50 cells.

**Figure S9.** **Wound healing.** Percent relative wound density (A), wound confluence (B) and wound width (C) measured upon treatment with **Pt^IV^P-5FUMeOBut** and **Pt^IV^56-5FUMeOBut** prodrugs, as well as 5FU, 5FUMeOBut and cisplatin in HT29 cells quantified every 4 h for up to 72 h**.**

**Figure S10. Cell wound healing post treatment.** Represenattive wound images at 0 and 72 h of treatemnte with **Pt^IV^P-5FUMeOBut** and **Pt^IV^56-5FUMeOBut** prodrugs, as well as 5FU, 5FUMeOBut ligands and cisplatin in HT29 cells. Incucyte^®^ phase contrast microscope used to collect bright-field live images using 10× objective.

**Figure S11.** Proteomic analysis of HT29 upon treatment with 5FU. A. principal component analysis. B. Number of DEPs. C. Volcano plot of DEPs significantly upregulated (red) and significantly downregulated (green) and non-significant (grey) proteins. Data points denote mean ± SEM. *n* = 3 from three separate experiments.

**Figure S12.** Proteomic analysis of HT29 upon treatment with 5FUMeOBut. A. principal component analysis. B. Number of DEPs. C. Volcano plot of DEPs significantly upregulated (red) and significantly downregulated (green) and non-significant (grey) proteins. Data points denote mean ± SEM. *n* = 3 from three separate experiments.

**Figure S13. Proteomic profiling of HT29 cells following cisplatin treatment.** A. principal component analysis. B. Number of DEPs in HT29 cells. C. Volcano plot showing DEPs, with upregulated proteins in red and downregulated proteins in green. Cisplatin data were obtained from previously reported experiments run in parallel. ^44^ Data points denote mean ± SEM. *n* = 3 from three independent experiments.

D

**Figure S14. Proteomic analysis of HT29 upon treatment with Pt^IV^P-5FUMeOBut.** A. principal component analysis. B. number of DEPs. C. volcano plot of DEPs upregulated proteins in red and downregulated proteins in green. D. pathway enrichment and gene act network analysis in most significance of dysregulated pathways. Data points denote mean ± SEM. *n* = 3 from three separate experiments.

 D

*.*

**Figure S15. Proteomic analysis of HT29 upon treatment with Pt^IV^56-5FUMeOBut.** A. principal component analysis. B. HT29 number of DEPs. C. volcano plot of DEPs upregulated proteins in red and downregulated proteins in green. D. pathway enrichment and gene act network analysis in most significance of dysregulated pathways. Data points denote mean ± SEM. *n* = 3 from three separate experiment.

**A**

| $\beta$-actin/ GAPDH normalised to control | **Control** | **Cisplatin** | **5FU** | **5FUMeOBut** | **Pt^IV^P-5FUMeOBut** | **Pt^IV^56-5FUMeOBut** |
| --- | --- | --- | --- | --- | --- | --- |
|  | 1 | 1.492 | 1.572 | 1.383 | 1.812 | 1.949 |

**B**

| $\beta$-tubulin/ GAPDH normalised to control | **Control** | **Cisplatin** | **5FU** | **5FUMeOBut** | **Pt^IV^P-5FUMeOBut** | **Pt^IV^56-5FUMeOBut** |
| --- | --- | --- | --- | --- | --- | --- |
|  | 1 | 0.833 | 0.849 | 0.889 | 0.551 | 0.418 |

**Figure S13.** *Cont.*

**C**

| $\alpha$-tubulin/ GAPDH normalised to control | **Control** | **Cisplatin** | **5FU** | **5FUMeOBut** | **Pt^IV^P-5FUMeOBut** | **Pt^IV^56-5FUMeOBut** |
| --- | --- | --- | --- | --- | --- | --- |
|  | 1 | 0.902 | 1.053 | 1.078 | 2.362 | 0.654 |

**Figure S13.** Full representative western blot of microtubule cytoskeleton protein markers in HT29. Represented data normalized to GAPDH relative to control. A. $\beta$-actin, B. $\beta$-tubulin and C. $\alpha$-tubulin. Protein expression upon treatment with 5FU, 5FUMeOBut, **P-5FUMeOBut** and **56-5FUMeOBut**, as well as cisplatin in HT29 cells at 72 h compared with control.

**A**

| p-AKT/AKT/GAPDH normalised to control | **Control** | **Cisplatin** | **5FU** | **5FUMeOBut** | **Pt^IV^P-5FUMeOBut** | **Pt^IV^56-5FUMeOBut** |
| --- | --- | --- | --- | --- | --- | --- |
|  | 1 | 0.519 | 1.148 | 0.835 | 0.492 | 0.580 |

**B**

| p-ERK/ERK/ GAPDH normalised to control | **Control** | **Cisplatin** | **5FU** | **5FUMeOBut** | **Pt^IV^P-5FUMeOBut** | **Pt^IV^56-5FUMeOBut** |
| --- | --- | --- | --- | --- | --- | --- |
|  | 1 | 0.691 | 1.060 | 0.840 | 0.709 | 0.820 |

**Figure S14.** *Cont.*

**C**

| p53/GAPDH  normalised to control | **Control** | **Cisplatin** | **5FU** | **5FUMeOBut** | **Pt^IV^P-5FUMeOBut** | **Pt^IV^56-5FUMeOBut** |
| --- | --- | --- | --- | --- | --- | --- |
|  | 1 | 1.369 | 1.703 | 1.728 | 2.804 | 1.871 |

**D**

| p21/GAPDH  normalised to control | **Control** | **Cisplatin** | **5FU** | **5FUMeOBut** | **Pt^IV^P-5FUMeOBut** | **Pt^IV^56-5FUMeOBut** |
| --- | --- | --- | --- | --- | --- | --- |
|  | 1 | 0.956 | 3.659 | 0.950 | 3.363 | 7.971 |

**Figure S14.** Full representative western blot of cell proliferation protein markers in HT29. Represented data normalised to GAPDH relative to control. A. p-AKT/AKT, B. p-ERK/ERK, C. p53 and D. p21.Protein expression upon treatment with 5FU, 5FUMeOBut, **P-5FUMeOBut** and **56-5FUMeOBut**, as well as cisplatin in HT29 cells at 72 h compared with control.

**A**

| Bax/GAPDH  normalised to control | **Control** | **Cisplatin** | **5FU** | **5FUMeOBut** | **Pt^IV^P-5FUMeOBut** | **Pt^IV^56-5FUMeOBut** |
| --- | --- | --- | --- | --- | --- | --- |
|  | 1 | 1.404 | 2.799 | 2.997 | 3.515 | 1.900 |

**Figure S15.** *Cont.*

**B**

| Bcl2/GAPDH  normalised to control | **Control** | **Cisplatin** | **5FU** | **5FUMeOBut** | **Pt^IV^P-5FUMeOBut** | **Pt^IV^56-5FUMeOBut** |
| --- | --- | --- | --- | --- | --- | --- |
|  | 1 | 0.432 | 1.192 | 1.259 | 0.499 | 0.633 |

**C**

| Cytochrome C/GAPDH  normalised to control | **Control** | **Cisplatin** | **5FU** | **5FUMeOBut** | **Pt^IV^P-5FUMeOBut** | **Pt^IV^56-5FUMeOBut** |
| --- | --- | --- | --- | --- | --- | --- |
|  | 1 | 0.749 | 1.472 | 1.671 | 2.145 | 1.435 |

**D**

| Procaspase 8/GAPDH  normalised to control | **Control** | **Cisplatin** | **5FU** | **5FUMeOBut** | **Pt^IV^P-5FUMeOBut** | **Pt^IV^56-5FUMeOBut** |
| --- | --- | --- | --- | --- | --- | --- |
|  | 1 | 1.080 | 1.388 | 0.765 | 0.134 | 0.503 |

**Figure S15.** *Cont.*

**E**

| Cleaved BID/GAPDH  normalised to control | **Control** | **Cisplatin** | **5FU** | **5FUMeOBut** | **Pt^IV^P-5FUMeOBut** | **Pt^IV^56-5FUMeOBut** |
| --- | --- | --- | --- | --- | --- | --- |
|  | 1 | 1.189 | 1.300 | 2.135 | 2.045 | 4.484 |

**F**

| Procaspase 9/GAPDH  normalised to control | **Control** | **Cisplatin** | **5FU** | **5FUMeOBut** | **Pt^IV^P-5FUMeOBut** | **Pt^IV^56-5FUMeOBut** |
| --- | --- | --- | --- | --- | --- | --- |
|  | 1 | 1.292 | 1.177 | 0.705 | 0.205 | 0.093 |

**G**

| Cleaved Caspase 3/GAPDH  normalised to control | **Control** | **Cisplatin** | **5FU** | **5FUMeOBut** | **Pt^IV^P-5FUMeOBut** | **Pt^IV^56-5FUMeOBut** |
| --- | --- | --- | --- | --- | --- | --- |
|  | 1 | 0.519 | 1.508 | 0.486 | 20.535 | 16.748 |

**Figure S15.** *Cont.*

**H**

|  | **Control** | **Cisplatin** | **5FU** | **5FUMeOBut** | **Pt^IV^P-5FUMeOBut** | **Pt^IV^56-5FUMeOBut** |
| --- | --- | --- | --- | --- | --- | --- |
| PARP-1 /GAPDH normalised to control | 1 | 1.271 | 1.516 | 1.483 | 0.548 | 0.196 |
| Cleaved PARP-1 /GAPDH normalised to control | 1 | 1.026 | 1.149 | 1.213 | 1.000 | 4.099 |

**Figure S15.** Full representative western blot of intrinsic and extrinsic apoptotic cell death markers in HT29. Represented data normalised to GAPDH relative to control. A. Bax, B. Bcl2, C. Cytochrome C, D.Procaspase 8, E. Cleaved BID, F. Procaspase 9, G. Procaspase 3 and H. PARP-1. Protein expression upon treatment with 5FU, 5FUMeOBut, **P-5FUMeOBut** and **56-5FUMeOBut**, as well as cisplatin in HT29 cells at 72 h compared with control.

**A**

| Beclin1/GAPDH  normalised to control | **Control** | **Cisplatin** | **5FU** | **5FUMeOBut** | **Pt^IV^P-5FUMeOBut** | **Pt^IV^56-5FUMeOBut** |
| --- | --- | --- | --- | --- | --- | --- |
|  | 1 | 1.675 | 1.719 | 1.816 | 3.159 | 1.721 |

**Figure S16.** *Cont.* **B**

| ATG5/GAPDH  normalised to control | **Control** | **Cisplatin** | **5FU** | **5FUMeOBut** | **Pt^IV^P-5FUMeOBut** | **Pt^IV^56-5FUMeOBut** |
| --- | --- | --- | --- | --- | --- | --- |
|  | 1 | 0.510 | 2.614 | 2.233 | 0.836 | 0.757 |

**C**

| ATG16L1/GAPDH  normalised to control | **Control** | **Cisplatin** | **5FU** | **5FUMeOBut** | **Pt^IV^P-5FUMeOBut** | **Pt^IV^56-5FUMeOBut** |
| --- | --- | --- | --- | --- | --- | --- |
|  | 1 | 0.741 | 0.752 | 1.459 | 0.552 | 1.133 |

**D**

| ATG9A/GAPDH  normalised to control | **Control** | **Cisplatin** | **5FU** | **5FUMeOBut** | **Pt^IV^P-5FUMeOBut** | **Pt^IV^56-5FUMeOBut** |
| --- | --- | --- | --- | --- | --- | --- |
|  | 1 | 1.295 | 0.513 | 0.761 | 2.501 | 2.414 |

**Figure S16.** *Cont.*

**E**

| ATG4B/GAPDH  normalised to control | **Control** | **Cisplatin** | **5FU** | **5FUMeOBut** | **Pt^IV^P-5FUMeOBut** | **Pt^IV^56-5FUMeOBut** |
| --- | --- | --- | --- | --- | --- | --- |
|  | 1 | 0.638 | 0.765 | 0.691 | 0.534 | 0.547 |

**F**

| LC3B/GAPDH  normalised to control | **Control** | **Cisplatin** | **5FU** | **5FUMeOBut** | **Pt^IV^P-5FUMeOBut** | **Pt^IV^56-5FUMeOBut** |
| --- | --- | --- | --- | --- | --- | --- |
|  | 1 | 5.108 | 2.379 | 2.133 | 2.053 | 3.802 |

**Figure S16.** Full representative western blot of autophagy markers in HT29. Represented data normalised to GAPDH relative to control. A. Beclin1, B. APG5L/ATG5, C. ATG16L1, D. ATG9A, E. ATG4B and F. LC3B. Protein expression upon treatment with 5FU, 5FUMeOBut, **Pt^IV^P-5FUMeOBut** and **Pt^IV^56-5FUMeOBut**, as well as cisplatin in HT29 cells at 72 h compared with control.

**Table S6. Antibody Concentrations used in Western Blot**

| **Supplier** | **Clone no.** | **Clone** | **Primary Antibody Target** | **Molecular Weight** |
| --- | --- | --- | --- | --- |
| Abcam | ab32503 | Rabbit monoclonal | BAX | 21 KDa |
| Abcam | ab196495 | Rabbit polyclonal | Bcl2 | 26 KDa |
| Abcam | ab32138 | Rabbit monoclonal | PARP-1 | pro-form 113 and 25 KDa (N-terminal catalytic domain) cleaved form of PARP1 |
| Abcam | ab133504 | Rabbit monoclonal | Cytochrome C | 11 or 14 KDa |
| Abcam | ab214430 | Rabbit monoclonal | Caspase 3 | 32 KDa and cleaved fragments at 17, 19 or 24 KDa |
| Abcam | ab108333 | Rabbit monoclonal | Caspase 8 | 55 KDa |
| Abcam | ab202068 | Rabbit monoclonal | Caspase 9 | 46, KDa |
| Abcam | ab8227 | Rabbit polyclonal | Beta Actin | 42 KDa |
| Abcam | ab7291 | Mouse monoclonal | Alpha Tubulin | 50 KDa |
| Abcam | ab6046 | Rabbit Polyclonal | Beta Tubulin | 50 KDa |
| Abcam | ab32389 | Rabbit monoclonal | p53 | 44 KDa |
| Abcam | ab109520 | Rabbit monoclonal | p21 | 21 KDa |
| Abcam | ab192591 | Rabbit polyclonal | p-ERK | 41-44 KDa |
| Abcam | ab184699 | Rabbit monoclonal | ERK | 42 KDa |
| Abcam | ab38449 | Rabbit polyclonal | p-AKT | 56 KDa |
| Abcam | ab8805 | Rabbit polyclonal | AKT | 56 KDa |
| Abcam | ab8245 | Mouse monoclonal | GAPDH | 36 or 40 KDa |
| Abcam | ab228668 | Rabbit polyclonal | APG5L/ATG5 | predicted at 32 KDA but seen at 55 KDa |
| Abcam | ab228525 | Rabbit polyclonal | ATG16L1 - N-terminal | 68-70 KDa |
|  |  |  | ATG4B | 44 KDa |
|  |  |  | ATG9A | 94-100 KDa |
|  |  |  | Beclin-1 | 52 KDa |
|  |  |  | LC3B | 14-16 KDa |
| Abcam | ab10640 | Rabbit polyclonal | Bid Cleavage Site | 15 KDa |

**References**

[1] Khoury, A., Elias, MG., Sakoff, J.A., Gilbert, J., Scott, K., Aldrich-Wright, J.R. Potent Anticancer 5-Fluorouracil Platinum(IV) Prodrugs. *Journal of Inorganic Biochemistry* **2025**.

[2] Elias, M. G., Fatima, S., Mann, T., Karan, S., Mikhael, M., Souza, P., Gordon, C.P, Scott, K. F., Aldrich-Wright, J.R. Anticancer Effect of PtIIPHENSS, PtII5MESS, PtII56MESS and their Platinum(IV)-dihydroxy derivatives Against Triple-Negative Breast Cancer and Cisplatin-Resistant Colorectal Cancer *Cancers* **2024**, *16* (Innovations in Cancer Drug Development Research). DOI: <https://doi.org/10.3390/cancers16142544>.
